# Antiaromatic character of cycloheptatriene-bis-annelated indenofluorene framework mainly originated from heptafulvene segment

**DOI:** 10.1038/s41598-018-35839-w

**Published:** 2018-12-05

**Authors:** Keitaro Yamamoto, Yutaka Ie, Norimitsu Tohnai, Fumitoshi Kakiuchi, Yoshio Aso

**Affiliations:** 1 The Institute of Scientific and Industrial Research (ISIR), Osaka University, 8-1 Mihogaoka, Ibaraki, Osaka 567-0047 Japan; 2 Japan Science and Technology (JST) Agency, ACT-C, 4-1-8 Honcho, Kawaguchi, Saitama 332-0012 Japan; 3 Department of Materials and Life Science, Graduate School of Engineering, Osaka University, 2-1 Yamadaoka, Suita, Osaka 565-0871 Japan; 4 Department of Chemistry, Faculty of Science and Technology, Keio University, 3-14-1 Hiyoshi, Kohoku-ku, Yokohama, Kanagawa 223-8522 Japan

## Abstract

Fully π-conjugated polycyclic hydrocarbons with antiaromatic character have attracted research attention because of their unique properties such as narrow energy gaps, and thus should find application as optical and electronic materials. Although antiaromatic 16π-electron frameworks can be constructed by the incorporation of multiple seven-membered rings in a fused fashion to install methylenecycloheptatriene (heptafulvene) segments, the development of corresponding benzo[1,2:4,5]di[7]annulene (BDA)-containing π-conjugated systems remains challenging due to the difficulty of their molecular design and synthesis. In this study, we develop an unprecedented chemical structure of cycloheptatriene-bis-annelated indenofluorene, which possesses both BDA and indenofluorene frameworks in a fused fashion. Physical measurements and X-ray analyses, along with theoretical calculations, indicated that antiaromaticity appeared in the BDA framework. By using the conjugated polycyclic hydrocarbon possessing both seven-membered and five-membered rings, this study provides fundamental insight into the strong antiaromatic nature of heptafulvene-based BDA framework.

## Introduction

**Figure 1:**
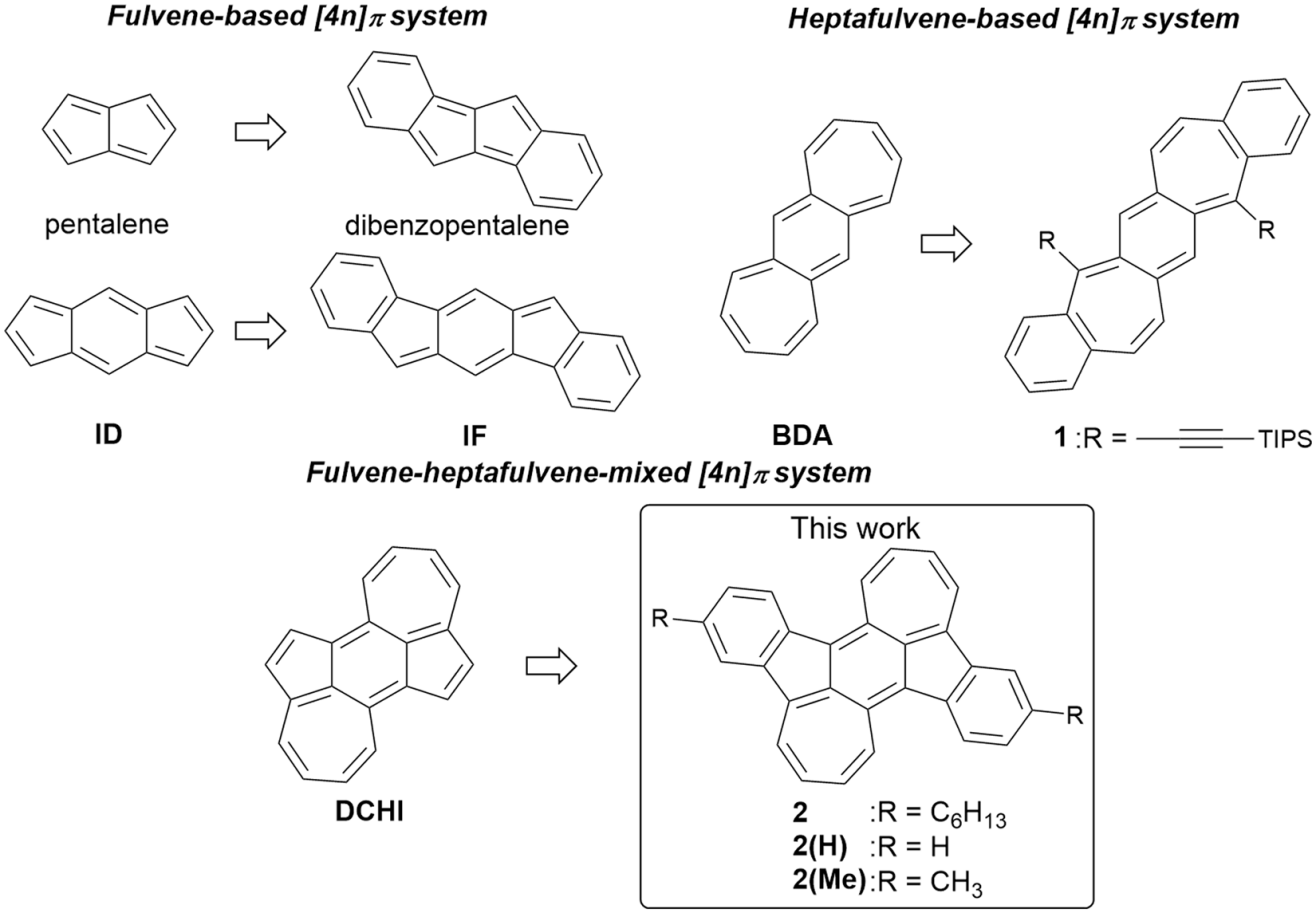
Chemical structures of representative antiaromatic compounds and target compound 2.

Increased interest has emerged in fully π-conjugated polycyclic hydrocarbons, which possess electronic properties desirable for potential applications as semiconducting materials in organic thin-film electronics^1–7^. While the majority of these systems are based on fused [4*n* + 2] π-electron aromatic molecules such as pentacene, the utilization of a [4*n*] π-electron antiaromatic character has emerged as a useful strategy to create new molecules with unusual electronic structures^8–11^. Since it is well known that the representative 8π- and 12π-electron frameworks pentalene and indacene (ID), respectively, are unstable due to the high antiaromatic character, the incorporation of methylenecyclopentadiene (fulvene) segments into an acene system has become a rational molecular design to construct [4*n*] π molecules. Indeed, dibenzopentalene^12–28^ and indenofluorene (IF)^29–37^ derivatives have been intensively developed in recent years (Fig. 1)^38,39^. These fulvene units can accept an electron to form stable aromatic cyclopentadienide anions and, thus, polycyclic fully conjugated hydrocarbons containing five-membered rings have intrinsically electron-accepting characteristics^31^. An alternative approach to access antiaromatic frameworks relies on the use of methylenecycloheptatriene (heptafulvene) segments to form tricyclic 16π-electron benzo[1, 2:4, 5]di[7]annulene (BDA), whereas pristine heptalene exhibits nonaromatic character due to its twisted non-planar geometry^40^. Although several polycyclic hydrocarbons containing a BDA framework have been synthesized^41–47^, the development of BDA-based antiaromatic compounds is still limited due to the difficulty of (1) the molecular design to incorporate heptafulvene units into the π-conjugated systems and (2) the synthetic method for the construction of seven-membered rings^41,42,44–46^. In contrast to the fulvene unit, the formation of cycloheptatrienyl cation by one-electron oxidation of heptafulvene leads to aromatic stabilization. Thus, the thin-film of 1 showed hole-transporting characteristics in organic field-effect transistor (OFET) devices^44–46^. However, irrespective of these electronically complemental properties, fundamental studies to directly investigate the antiaromatic character between the ID and BDA frameworks have not been carried out so far. Therefore, we sought to construct an unprecedented π-conjugated polycyclic hydrocarbons by introducing multiple five-membered and seven-membered rings together in a conjugated molecule. Based on this strategy, we combined ID and BDA, resulting in the molecular framework of dicyclohepta[*cd*,*ij*]-*s*-indacene (DCHI), and synthesized the benzene-fused cycloheptatriene-bis-annelated indenofluorene 2 (Fig. 1). Herein, we describe the synthesis, structure, properties, and OFET characteristics of 2.

## Results and Discussion

**Figure 2:**
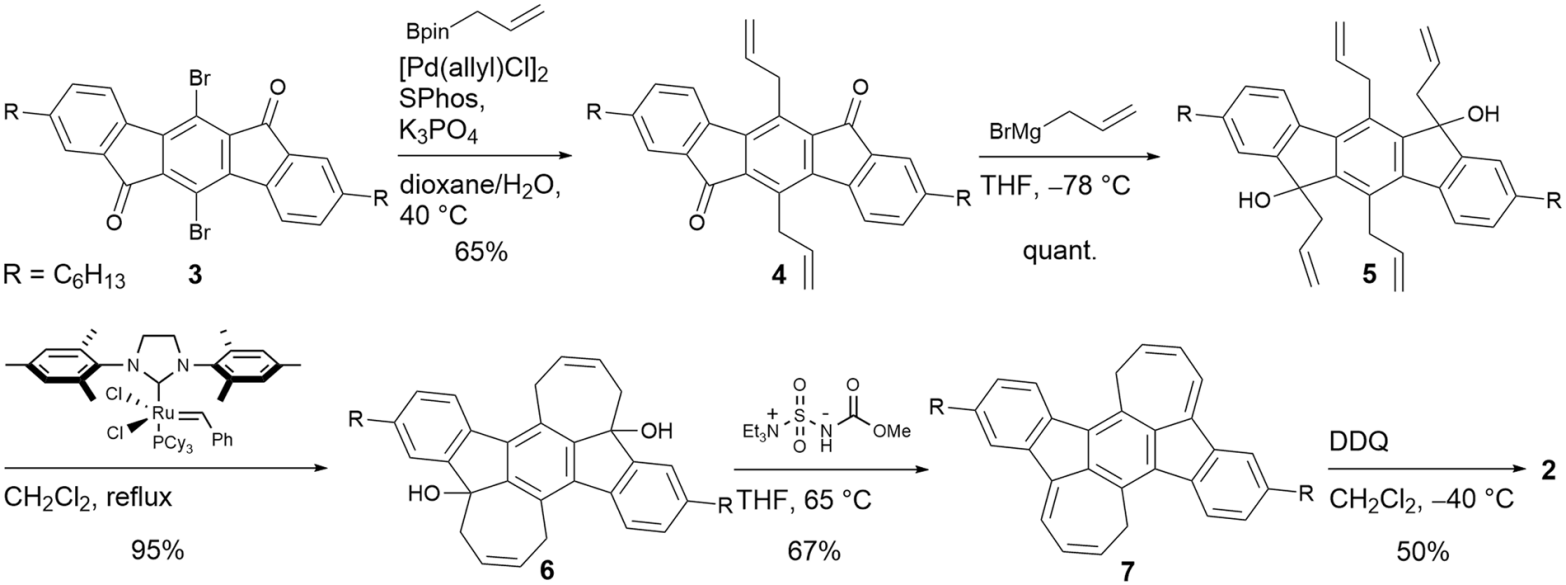
Synthetic route of 2.

Although the formation of the DCHI framework by photochemical reaction of *o*-diethynylbenzene derivatives was previously inferred^48^, it was disproven based on theoretical calculations of the spectroscopic data^49^ and reconsidered^50^. Therefore, the synthesis of this framework was heretofore unknown. With these precedents in mind, we used an olefin metathesis reaction as a key step to construct the seven-membered ring framework. The synthetic route to 2 starting from compound 3 is shown in Fig. 2. The Suzuki coupling reaction between 3 and allylboronic acid pinacol ester in the presence of [Pd(allyl)Cl]_2_ afforded di-allylated compound 4, which was reacted with allylmagnesium bromide to give the tetra-allylated compound 5. The construction of seven-membered rings was achieved by olefin metathesis using the second generation Grubbs’ catalyst to give key intermediate 6 in 95% yield^51^. We here report that compounds 5 and 6 were isolated as a mixture of diastereomers. Then, treatment of 6 with the Burgess reagent provided dehydrated compound 7^52^, and subsequent oxidation with 2,3-dichloro-5,6-dicyano-*p*-benzoquinone (DDQ) produced the target compound 2 in 50% yield. Note that the presence of the hexyl group is essential to impart enough solubility of 2 to enable characterization by NMR and other spectroscopic measurements. Detailed synthetic procedures and characterization data of the new compounds are summarized in the Methods section. The NMR spectra of 2 are shown in Supplementary Figs 1 and 2. As shown in Supplementary Fig. 2, the ^1^H NMR spectrum of 2 in CDCl_3_ showed a downfield shift of the benzene protons by 0.42–0.62 ppm as compared to the indenofluorene derivative IF(Me)-TA (structure shown in Supplementary Fig. 2), indicating the increased contribution of aromatic character in the benzene ring for 2. The proton signals in the seven-membered ring were observed in the upfield region between 5.95 and 7.29 ppm. Since the ^1^H NMR signals for the seven-membered ring of the antiaromatic BDA framework are reported to appear in a similar δ range of 4.88–6.30 ppm^41^, contribution of the antiaromatic character in the seven-membered ring of 2 is expected.

Thermogravimetric analysis (TGA) of 2 showed a 5% weight-loss temperature of 443 °C under a nitrogen atmosphere (Supplementary Fig. 3(a)). The differential scanning calorimetry (DSC) profile of 2 showed endothermic and exothermic peaks at 234 and 250 °C, respectively, during the first heating process (Supplementary Fig. 3(b)). On the other hand, no clear peaks were detected during the second heating process, and an insoluble black solid was observed after the DSC measurements. Given that only a slight weight loss was seen in TGA up to 443 °C, we considered that 2 melted at 234 °C, and intermolecular reactions occurred in the melting state to give unidentifiable insoluble products.

**Figure 3:**
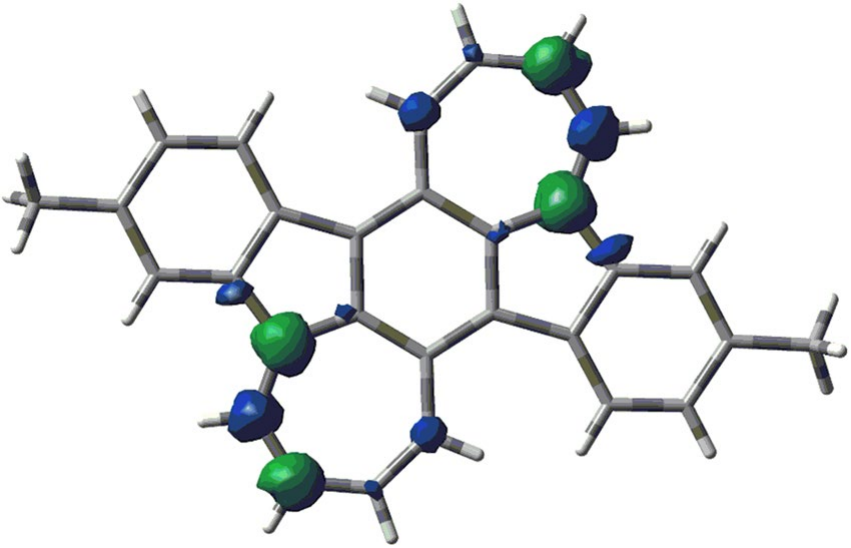
Spin density distribution of 2(Me) calculated at the UB3LYP/6-31 G(d) level.

Since the indenofluorene derivatives include a *p*-quinodimethane core, it is known that these molecules show biradical character originating from the aromatization of the central benzene ring^53^. Thus, the singlet biradical characters (*y*) of 2(Me) and IF were estimated using the occupation numbers of the spin-unrestricted Hartree–Fock natural orbitals. As a result, 2(Me) displayed a moderate singlet biradical character (*y = *0.49), which is larger than that of IF (*y* = 0.30). Since the calculated spin density for 2(Me) shows the largest amplitude at the fused carbons between the five- and seven-membered rings and is distributed to the seven-membered ring (Fig. 3), the biradicaloid electronic structure is thought to be stabilized by the spin delocalization at the seven-membered rings. The theoretically estimated singlet-triplet energy gap (Δ*E*_S-T_ ≡ *E*_T_ − *E*_S_) of 2(Me) at the UB3LYP/6-31 G(d,p) level is a large positive value of 92.5 kJ mol^−1^, indicating that the biradical structure of 2 has an exclusively singlet nature, which is consistent with the observation of sharp ^1^H NMR signals (Supplementary Fig. 1).

**Figure 4:**
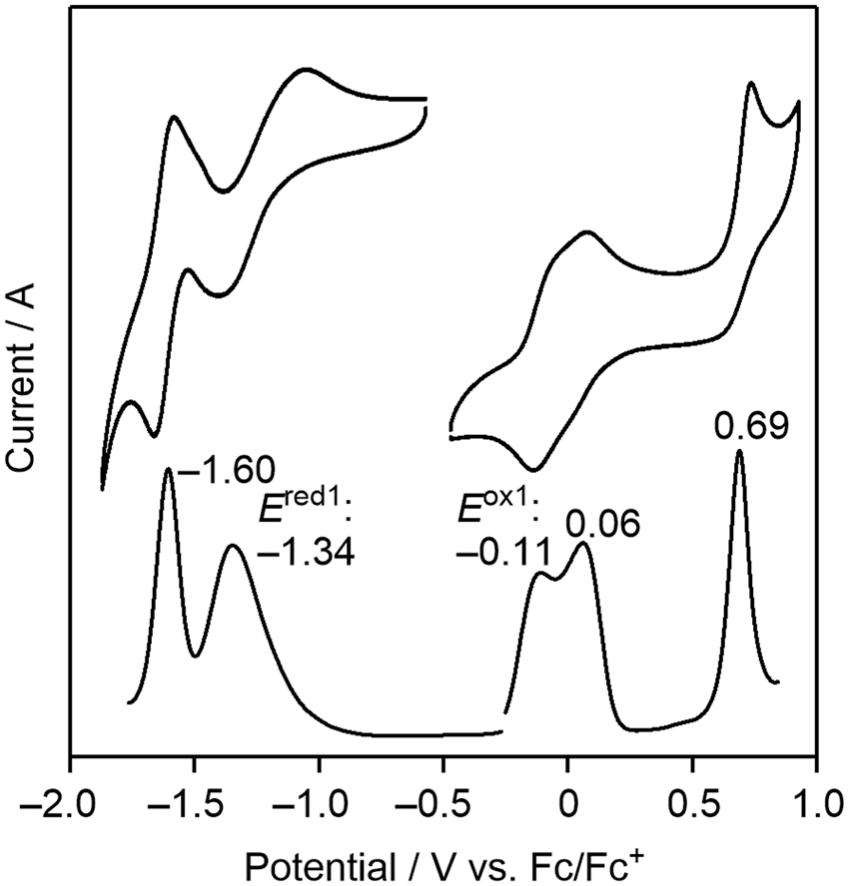
CV (top) and DPV (bottom) of 2 in CH_2_Cl_2_ containing 0.1 M TBAPF_6_.

**Figure 5:**
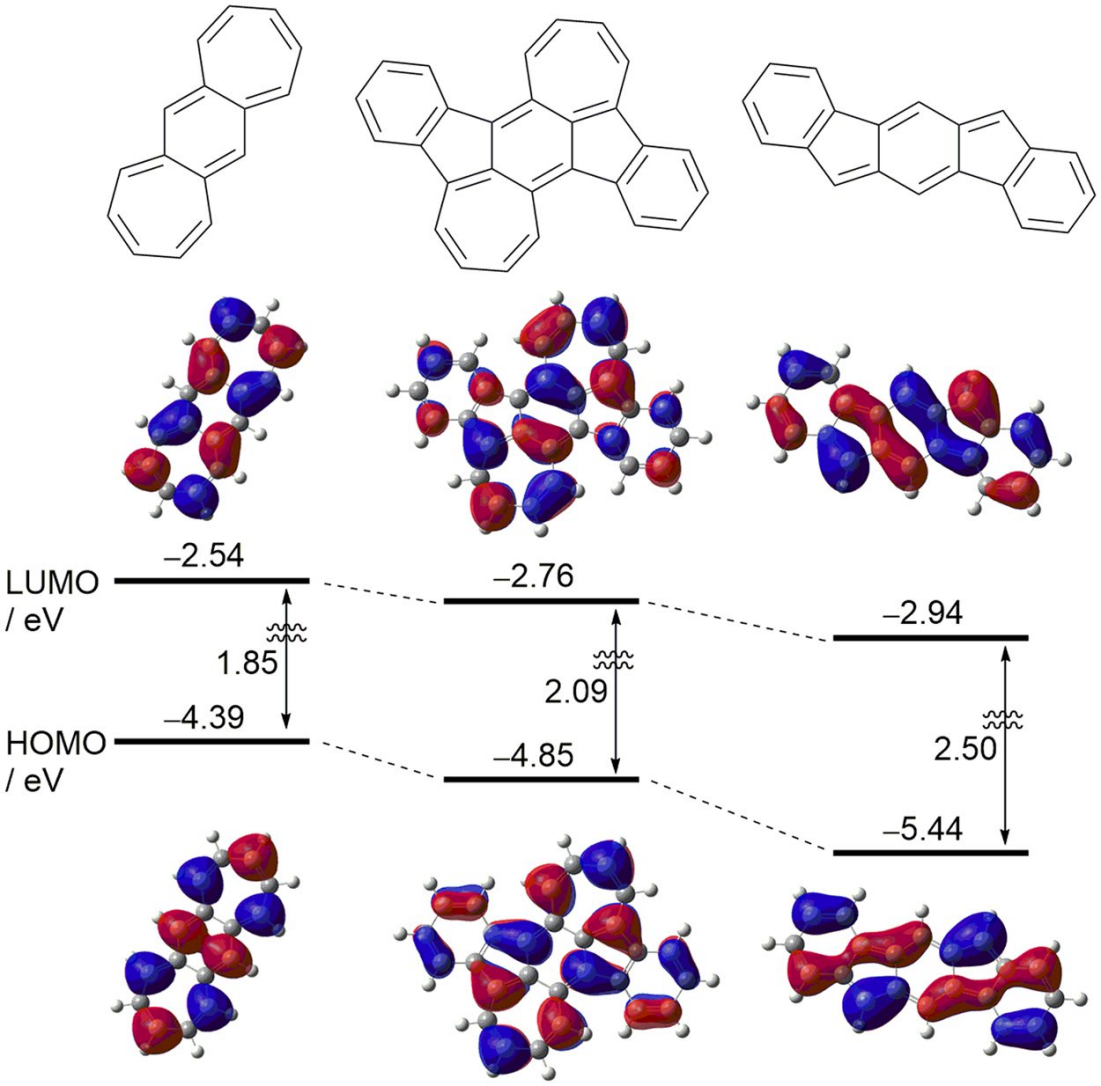
Energy levels and molecular orbitals of BDA (left), 2(H) (center), and IF (right) calculated at B3LYP/6-311 + G(d,p) level.

The electrochemical behavior of 2 was investigated by cyclic voltammetry (CV) and differential pulse voltammetry (DPV) measurements in CH_2_Cl_2_ containing 0.1 M tetrabutylammonium hexafluorophosphate (TBAPF_6_) as a supporting electrolyte. All potentials were calibrated against a ferrocene/ferrocenium (Fc/Fc^+^) couple as the standard. As shown in Fig. 4, the CV of 2 revealed three oxidation and two reduction processes, and the redox potentials were determined from DPV. From the first oxidation potential (*E*^ox1^) and first reduction potential (*E*^red1^) and the assumption that the energy level of Fc/Fc^+^ is −4.8 eV below the vacuum level^54–56^, the highest occupied molecular orbital (HOMO) and lowest unoccupied molecular orbital (LUMO) energy levels (*E*_HOMO_ and *E*_LUMO_) of 2 were estimated to be −4.69 and −3.46 eV, respectively. Based on these values, the HOMO-LUMO energy gap of 2 is calculated to be 1.23 eV. Interestingly, these *E*_HOMO_ and *E*_LUMO_ values are significantly different from those of IF(Me)-TA (*E*_HOMO_ = −5.84 eV and *E*_LUMO_ = −3.99 eV)^30^. In order to understand the origin of this phenomenon, density functional theory (DFT) calculations of 2(H), IF, and BDA at the B3LYP/6-311 + G(d, p) level were performed. As shown in Fig. 5, the theoretically estimated *E*_HOMO_ and *E*_LUMO_ values of 2(H) and IF qualitatively mimic the experimental values. Although the HOMO and LUMO orbitals of 2(H) are delocalized over the π-conjugated backbones, both the *E*_HOMO_ and *E*_LUMO_ values of 2(H) are between those of BDA and IF and closer to those of BDA. These results indicate that the electronic structure of the 2(H) molecule is formed from a hybrid of those of BDA and IF, and that the contribution of the electronic character of BDA seems to be dominant.

**Figure 6:**
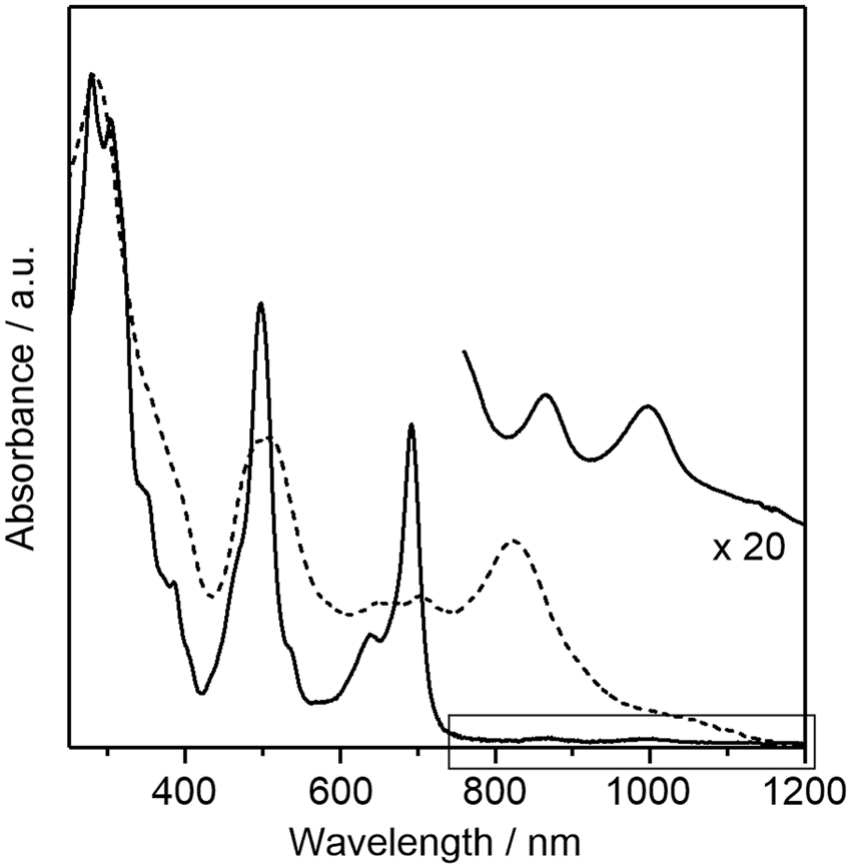
UV-vis-NIR absorption spectra of 2 in CH_2_Cl_2_ (solid line) and thin-films (dashed line). The magnified spectrum in CH_2_Cl_2_ between 800 and 1200 nm is also shown.

To investigate the photophysical properties, a UV-vis-NIR absorption measurement of 2 in a CH_2_Cl_2_ solution was performed. As shown in Fig. 6, the absorption spectrum of 2 includes three intense bands with absorption maxima (*λ*_max_) at 279 (ε = 55000 M^−1^ cm^−1^), 498 (36000 M^−1^ cm^−1^), and 692 (26000 M^−1^ cm^−1^) nm and weak bands at 866 and 997 nm. A time-dependent (TD)-DFT calculation at the B3LYP/6-31 G (d,p) level indicated that these intense bands were mainly assigned to HOMO − 1 to LUMO + 1, HOMO to LUMO + 2, and HOMO to LUMO transitions, respectively (see the Electronic Supplementary Information). Considering that 2(Me) possesses a moderate singlet biradical character (Fig. 3), the weak bands in the NIR region are assignable to the double excitation state of an open biradicaloid electronic structure^57,58^. Compared to the solution spectra, a new broad band was observed in the thin-films. This phenomenon is attributed to the intermolecular electronic interactions of π−π stacked backbones, which is favorable for carrier transport in thin-film devices (discussed later).

**Figure 7:**
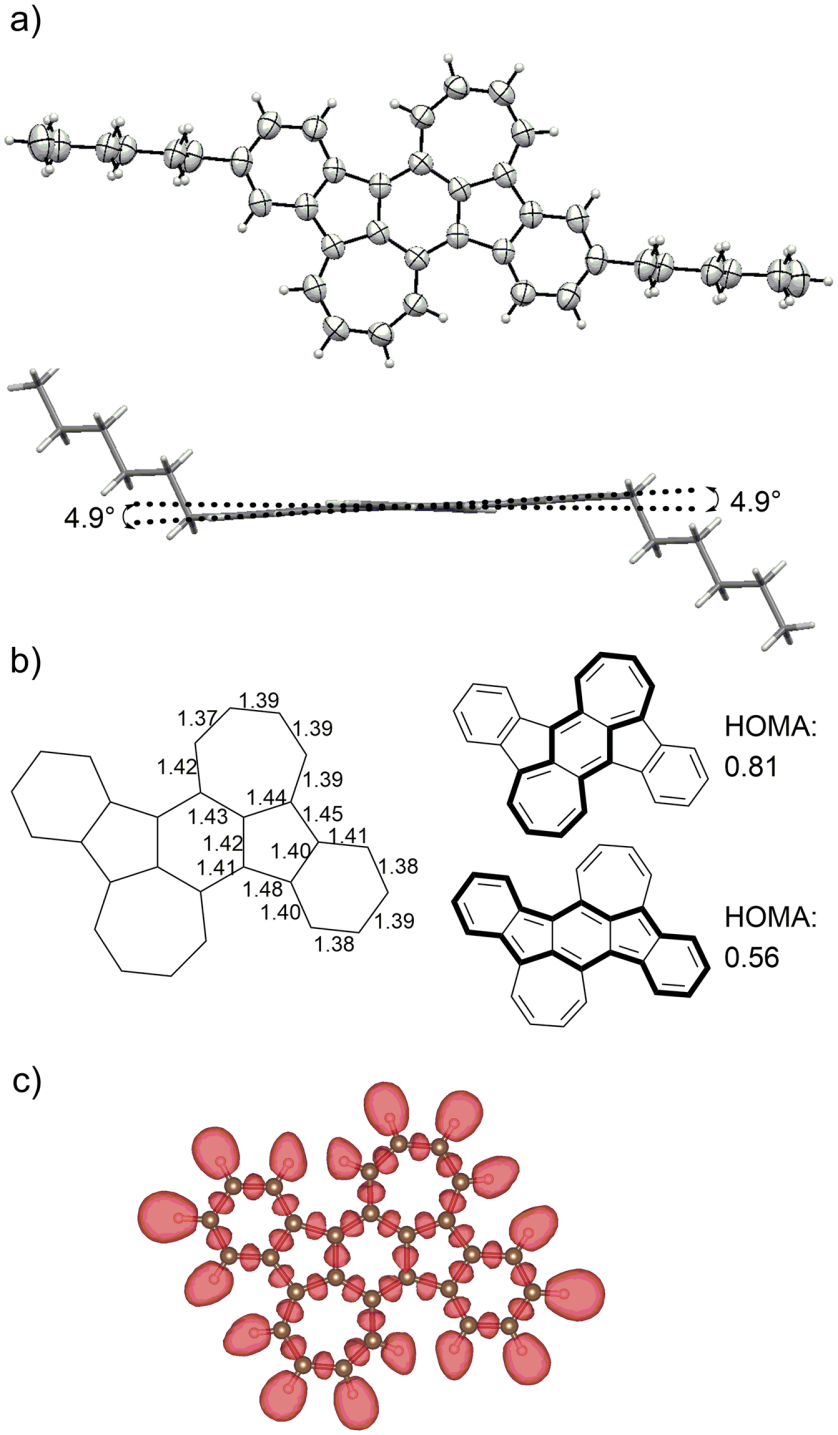
(**a**) ORTEP drawing of 2 for top view and side view; (**b**) bond lengths for the crystal structure (left) and HOMA values for BDA and IF frameworks. (**c**) Isosurface plot of 2 at an isosurface level of 0.73.

The molecular structure of 2 was unambiguously determined using X-ray crystallographic analysis of crystals grown by the slow evaporation of hexane/CHCl_3_ solutions. As shown in Fig. 7(a), the π-conjugated framework of compound 2 holds a nearly planar structure: the deviations of carbon atoms constituting the BDA core from the mean plane of this core are less than 0.04 Å, and the dihedral angles between the mean planes of central BDA and the outer benzene rings are 4.9°, as shown in Fig. 7(a). The structure of 1 was reported to show clear bond alternation in the *p*-quinodimethane core due to the increased contribution from resonance form 1a (structure shown in Supplementary Fig. 4), which in turn is attributed to the presence of the outer fused benzene rings^44^. On the other hand, the extent of the bond alternation for the BDA framework in 2 was rather small, with C-C bond lengths varying within the range of 1.37–1.44 Å (Fig. 7(b)). To further assess the degree of bond alternation, we calculated the harmonic oscillator model of aromaticity (HOMA) values^59^ based on the reported X-ray information. As shown in Supplementary Fig. 5, the HOMA value of the BDA framework in 1 was determined to be 0.31. In contrast, the HOMA value of the BDA framework in 2 was calculated to be as high as 0.81, supporting the small degree of bond alternation and resulting delocalized electronic structure. A similar trend was also observed for the antiaromatic ID framework: the absence of fused benzene rings in compound A leads to an increase in the HOMA value (Supplementary Fig. 5). These results indicate that pristine antiaromatic frameworks show large HOMA values; thus, the BDA framework in 2 is expected to show antiaromatic character. Interestingly, this HOMA value is larger than that of the indenofluorene framework in 2 (0.56), indicating that the contribution of 16π (BDA) and 6π × 2 (two outer benzene rings) is larger than that of 20π (indenofluorene) in the electronic structure of 2. To investigate the electronic contribution of the π electrons in 2, we performed an electron localization function (ELF) estimation^60^. The ELF isosurface plot (Fig. 7(c)) showed that the π electron pairs are uniformly distributed over the entire molecule. This result clearly indicates that the π-conjugation is delocalized over the entire π conjugated framework in 2, despite the different HOMA values of BDA and IF frameworks.

**Figure 8:**
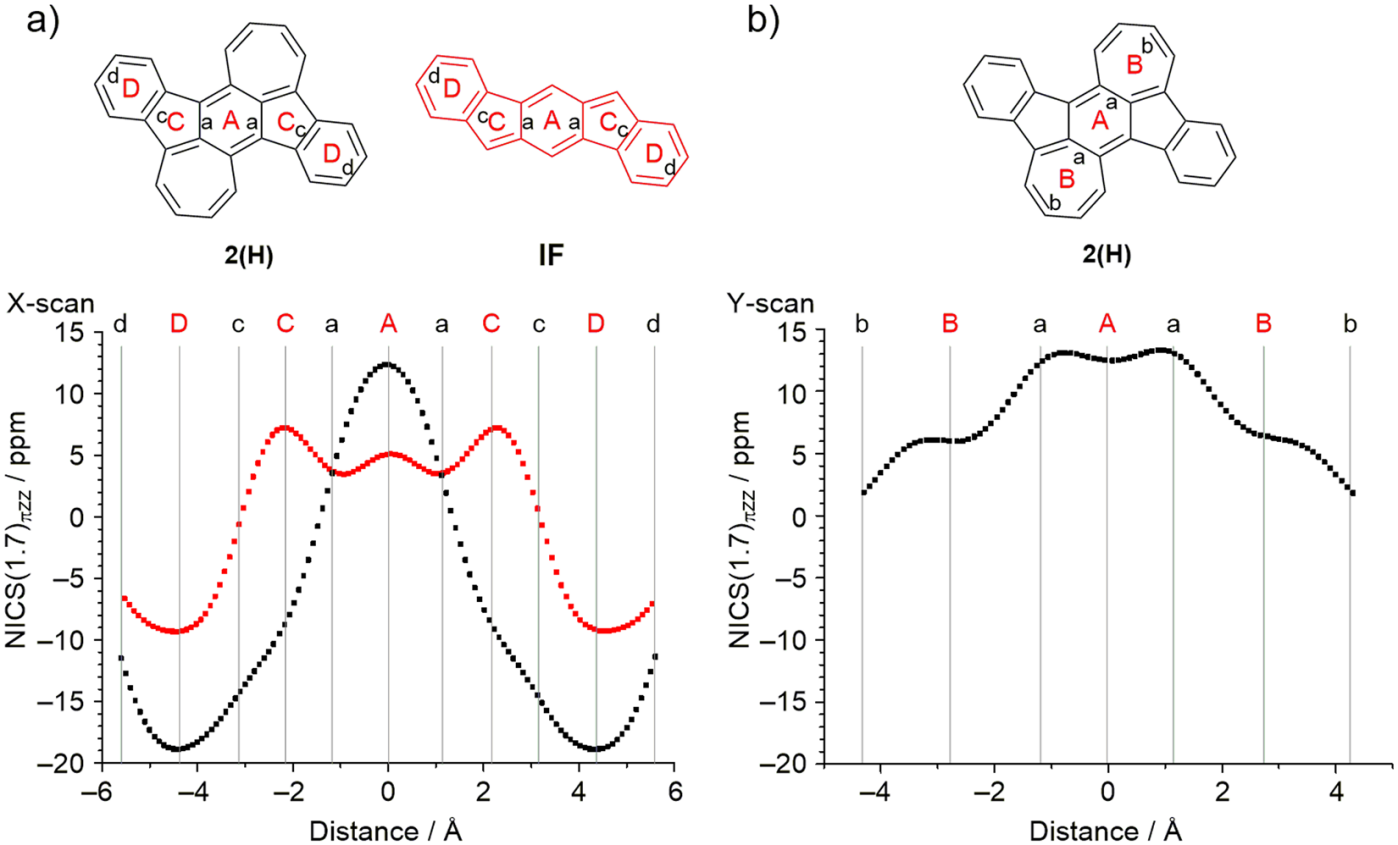
(**a**) NICS(1.7)_πzz_-*X*-scans for 2(H) (black) and IF (red) and (**b**) NICS(1.7)_πzz_-*Y*-scans for 2(H).

**Figure 9:**
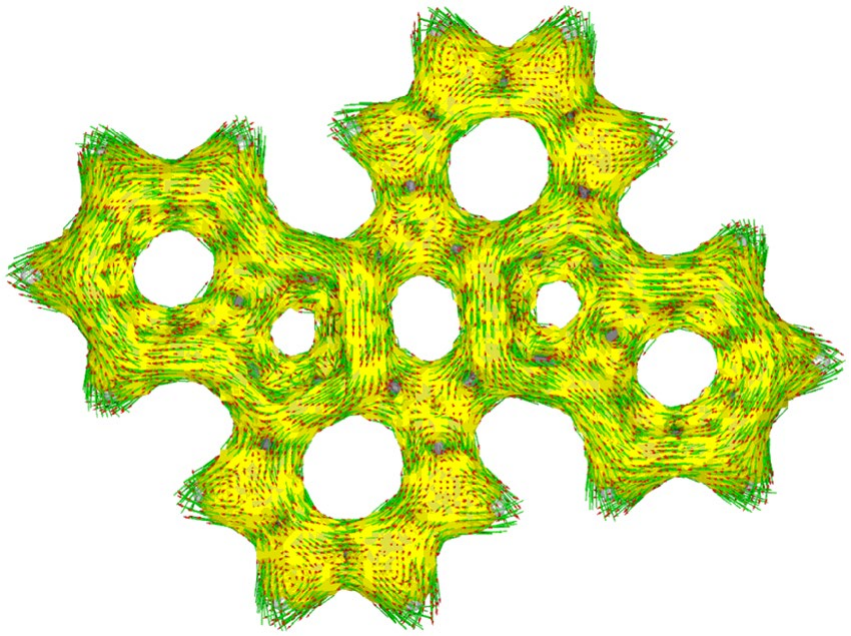
ACID-derived induced ring current map for 2.

Based on this molecular structure, we estimated the aromaticity of the π-conjugated framework of 2(H) by the nucleus independent chemical shift (NICS). The results of the NICS(1.7)_πzz_-*XY*-scans^61^ are summarized in Fig. 8. The NICS(1.7)_πzz_ values of 2(H), calculated at the GIAO-B3LYP/6-311 + G(d) level, are *δ* = +12.40, +6.19, −9.48, and −18.90 ppm for the rings A, B, C, and D, respectively. The positive values of rings A and B indicate that the BDA framework has an antiaromatic character. On the other hand, the indenofluorene core composed of rings C and D in 2(H) exhibits an aromatic character, which is in contrast with the result of pristine indenofluorene IF, in which the five-membered ring shows an antiaromatic character^33^. Since framework 2(H) contains both five- and seven-membered rings, this calculated aromatic character of the C ring is considered to result from increased electron density owing to electrical polarization between these rings. This was confirmed by the calculation of the electrostatic potential of 2(H), which was similar to that calculated for benz[*a*]azulene, as shown in Supplementary Fig. 6. These results are in agreement with the aforementioned findings based on the HOMA values, indicating the large electronic contribution of the BDA framework. To further support the antiaromaticity of the BDA framework in 2, we conducted anisotropy of the current-induced density (ACID) analysis^62^. Compound 2 showed continuous paratropic ring currents in the BDA framework (Fig. 9). On the other hand, diatropic ring currents were seen in the C_5_-C_6_ frameworks. This result clearly indicates that 2 has local antiaromaticity in the BDA framework and local aromaticity in the C_5_-C_6_ frameworks, consistent with the results of NICS calculation.

In the molecular packing diagram, 2 takes a herringbone π-stacked motif with minimum intermolecular π–π distances of 3.42 Å (Supplementary Fig. 7(a)). On the basis of the calculation by the Amsterdam Density Functional (ADF) program at the PW91/TZP level, the transfer integrals for hole transport (*t*_HOMO_) and electron transport (*t*_LUMO_) between adjoining molecules were estimated. As summarized in Supplementary Fig. 7(b), 2 showed large *t*_HOMO_ and *t*_LUMO_ of 91.4 and 111.6 meV, respectively, between facial-stacked molecules, and, thus, the construction of charge-carrier transporting pathways is expected along the stacking direction.

**Figure 10:**
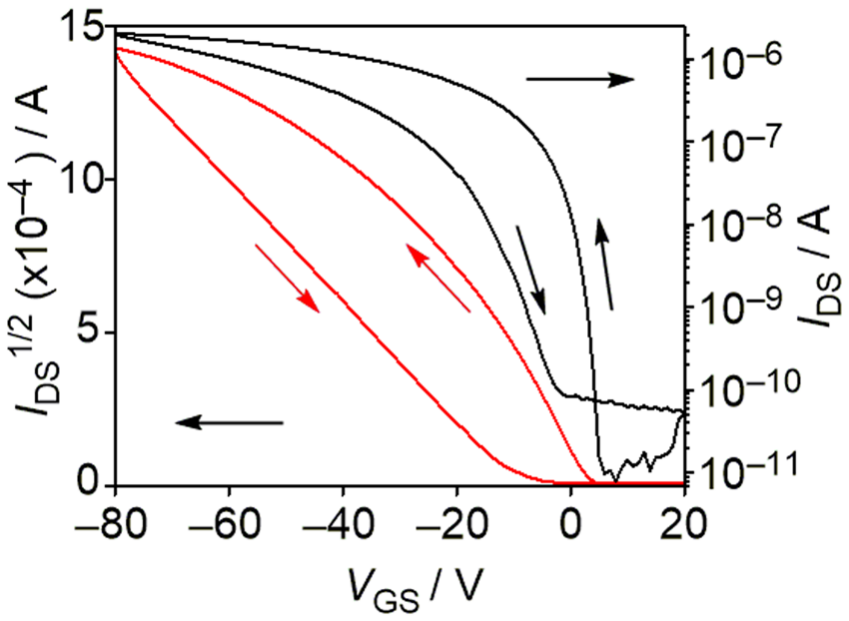
OFET transfer characteristics of thin-film based on 2. *I*_DS_ and *V*_GS_ denote source-drain current and gate voltage, respectively.

Reflecting the high thermal stability of 2, the thin-films for OFET measurements could be prepared by vacuum deposition onto hexamethyldisilazane (HMDS)-modified Si/SiO_2_ substrates. The atomic force microscopy (AFM) image of this film exhibited interconnected micrometer-sized grains (Supplementary Fig. 8(a)). X-ray diffraction (XRD) of the thin-film showed clear diffractions, indicating the formation of crystalline structures in thin-films. According to the X-ray crystal structure (Supplementary Fig. 7(a)), the peak at 2*θ*  = 5.2° can be indexed as a (001) diffraction peak with a *d* spacing of 17.0 Å, implying that the molecules are aligned with the crystal *c*-axis perpendicular to the SiO_2_ surface. To evaluate the charge-transport characteristics of the thin-films, OFET devices with bottom-gate bottom-contact configuration were fabricated. As shown in Fig. 10, this device showed hole-transporting characteristics with a field-effect hole mobility of 3.0 × 10^−5^ cm^2^ V^−1^ s^−1^ with a current on/off ratio of 10^5^. On the other hand, electron-transporting behavior was not observed for 2. This p-type response is explained by the high-lying HOMO energy level of 2. Importantly, taking the intrinsic electron-transporting behavior of the indenofluorene chromophore into consideration, embedding the BDA framework significantly influences the type of charge carriers. This trend is in good agreement with the estimated antiaromaticity of the BDA framework.

## Summary

In conclusion, in order to directly investigate the antiaromatic character between the ID and BDA frameworks, we successfully synthesized new polycyclic hydrocarbon 2, which contains a fused dicyclohepta[*cd*,*ij*]-*s*-indacene framework in the molecule. Electrochemical and photophysical measurements revealed that 2 has a relatively high HOMO energy level and a narrow HOMO-LUMO energy gap, and these properties mainly come from the BDA framework. Investigation of the X-ray crystal structure and theoretical calculation indicates that the contribution of antiaromatic character for the BDA framework is dominant in the molecule. These results clearly demonstrate that the unique character of 2 originated from the heptafulvene-based BDA framework, and we can conclude that the development of new antiaromatic compounds possessing the DCHI or BDA frameworks will pave the way to the fundamental understanding of antiaromaticity. Considering that 2 shows hole-transporting characteristics, fine-tuning of molecular design would aid the development of high-performance electronic materials. Further investigation on the development of such compounds to reveal the structure-property-semiconducting performance relationship is currently underway in our group.

## Methods

### General Information

Column chromatography was performed on silica gel. KANTO Chemical silica gel 60N (40–50 μm). Thin-layer Chromatography (TLC) plates were visualized with UV light. Preparative gel-permeation chromatography (GPC) was performed on a Japan Analytical LC-918 equipped with JAI-GEL 1H/2H. ^1^H and ^13^C NMR spectra were recorded on a JEOL JNM-ECS400 or JEOL JNM-ECA600 spectrometer in CDCl_3_ with tetramethylsilane (TMS) as an internal standard. Data are reported as follows: chemical shift in ppm (*δ*), multiplicity (s = singlet, d = doublet, t = triple, m = multiplet, br = broad), coupling constant (Hz), and integration. UV-vis-NIR spectra were recorded on a Shimadzu UV-3600 spectrophotometer. All spectra were obtained in spectrograde solvents. TGA and DSC were performed under nitrogen at a heating rate of 10 °C min^−1^ with a Shimadzu TGA-50 and a Shimadzu DSC-60, respectively. Cyclic voltammetry was carried out on a BAS CV-620C voltammetric analyzer using a platinum disk as the working electrode, platinum wire as the counter electrode, and Ag/AgNO3 as the reference electrode at a scan rate of 100 mV s^−1^. High-resolution mass spectrum (HRMS) was obtained atmospheric pressure chemical ionization (APCI) or electrospray ionization (ESI) methods using a Thermo scientific LTQ Orbitrap XL. Elemental analyses were performed on PerkinElmer LS-50B by the elemental analysis section of the Comprehensive Analysis Center (CAC) of ISIR, Osaka University. The surface structures of the deposited organic film were observed by atomic force microscopy (Shimadzu, SPM9600), and the film crystallinity was evaluated by an X-ray diffractometer (Rigaku, SmartLab). X-ray diffraction patterns were obtained using Bragg-Brentano geometry with CuK*α* radiation as an X-ray source with an acceleration voltage of 45 kV and a beam current of 200 mA. The scanning mode was set to 2*θ*–*θ* scans between 2°–30° with scanning steps of 0.01°.

### Synthetic information

Unless stated otherwise, all reagents were purchased from commercial sources and used without purification. Synthetic procedure of 3 was shown in Supplementary Fig. 9, and the corresponding characterization data was summarized in the Electronic Supplementary Information.

### Synthesis of 4

Compound 3 (200 mg, 0.329 mmol), allylboronic acid pinacol ester (0.240 mL, 1.32 mmol), allylpalladium(II) chloride dimer (24.0 mg, 0.0658 mmol), SPhos (108 mg, 0.263 mmol), and K_3_PO_4_ (350 mg, 1.65 mmol), 1,4-dioxance (7.0 mL) and water (1.9 mL) were added to a reaction vial. The vial was purged with N_2_. After stirring for 4 h at 40 °C, water was poured into the reaction mixture. The resultant mixture was extracted with ethyl acetate (EtOAc) and the combined organic layer was washed with brine. After drying with MgSO_4_, the solvent was removed under reduced pressure and the residue was purified by column chromatography on silica gel (hexane: CHCl_3 = _2: 1) to give 4 (113 mg, 65%) as a magenta solid. ^1^H NMR (400 MHz, CDCl_3_, TMS): *δ* 0.89 (t, *J* = 6.9 Hz, 6 H), 1.27–1.38 (m, 12 H), 1.56–1.65 (m, 4 H), 2.66 (t, *J* = 7.6 Hz, 4 H), 4.24 (d, *J* = 5.0 Hz, 4 H), 5.08–5.13 (m, 4 H), 6.05–6.16 (m, 2 H), 7.30 (dd, *J* = 7.8, 1.8 Hz, 2 H), 7.47 (d, *J* = 1.8 Hz, 2 H), 7.57 (d, *J* = 7.8 Hz, 2 H); ^13^C NMR (150 MHz, CDCl_3_): *δ* 14.1, 22.6, 28.9, 30.3, 31.1, 31.7, 35.6, 116.4, 123.6, 124.0, 133.9, 134.3, 134.9, 134.9, 136.1, 140.2, 144.1, 145.5; HRMS (*m*/*z*): [M + H]^+^ calcd. for C_38_H_43_O_2_, 531.3258; found, 531.3248.

### Synthesis of 5

To a solution of 4 (190 mg, 0.358 mmol) in THF (23 mL), 1.0 M allylmagnesium bromide solution in Et_2_O (0.788 mL, 0.788 mmol) was added at −78 °C. After stirring for 1 h at −78 °C, the reaction was quenched by pouring saturated NH_4_Cl aqueous solution. The combined organic was extracted with EtOAc and washed with water. After drying over with MgSO_4_, the solvent was removed under reduced pressure to give 5 (220 mg, quant.) as an orange solid. ^1^H NMR (600 MHz, CDCl_3_, TMS): *δ* 0.89 (t, *J* = 6.9 Hz, 6 H), 1.27–1.38 (m, 12 H), 1.59–1.68 (m, 4 H), 2.19–2.23 (m, 2 H), 2.61–2.69 (m, 4 H), 2.92–3.07 (m, 4 H), 3.95–4.01 (m, 1 H), 4.08–4.14 (m, 1 H), 4.30–4.36 (m, 1 H), 4.42–4.47 (m, 1 H), 4.65–4.77 (m, 4 H), 4.86–4.96 (m, 2 H), 5.06–5.16 (m, 4 H), 6.14–6.23 (m, 2 H), 7.11–7.15 (m, 2 H), 7.32 (s, 2 H), 7.54–7.60 (m, 2 H); ^13^C NMR (150 MHz, CDCl_3_): *δ* 14.1, 22.6, 28.9, 29.0, 31.5, 31.8, 31.9, 35.9, 36.0, 43.7, 43.8, 82.7, 116.1, 116.2, 117.9, 118.0, 122.8, 123.0, 123.4, 123.5, 128.8, 128.9, 129.5, 129.9, 132.5, 132.6, 136.0, 136.2, 136.4, 138.7, 138.8, 142.1, 147.3, 147.5, 149.8; HRMS (*m*/*z*): [M + Na]^+^ calcd. for C_44_H_54_O_2_Na, 637.4016; found, 637.4014.

### Synthesis of 6

Grubbs catalyst (22.4 mg, 0.0264 mmol) was added to a solution of 5 (203 mg, 0.330 mmol) in CH_2_Cl_2_ (8.0 mL), and the reaction was refluxed. After stirring for 2 h, the reaction mixture was filtrated through pad of celite. The solvent was removed under reduced pressure, and the residue was purified by column chromatography on silica gel (CHCl_3_) to give 6 (175 mg, 95%) as a brown solid. ^1^H NMR (600 MHz, CDCl_3_, TMS): *δ* 0.89 (t, *J* = 6.5 Hz, 6 H), 1.28–1.40 (m, 12 H), 1.62–1.69 (m, 4 H), 2.30–2.40 (m, 4 H), 2.66 (t, *J* = 7.6 Hz, 4 H), 2.98–3.04 (m, 2 H), 4.09–4.21 (m, 4 H), 5.68–5.74 (m, 2 H), 5.89–6.00 (m, 2 H), 7.16–7.19 (m, 2 H), 7.34–7.37 (m, 2 H), 7.73–7.81 (m, 2 H); ^13^C NMR (150 MHz, CDCl_3_): *δ* 14.1, 22.6, 28.4, 28.9, 29.1, 31.5, 31.7, 35.9, 38.8, 38.9, 80.9, 81.0, 123.0, 123.1, 123.2, 125.5, 125.9, 126.1, 126.4, 129.1, 131.1, 131.4, 135.0, 135.1, 136.7, 136.8, 142.4, 142.5, 148.5, 148.9, 150.8, 150.9; HRMS (*m*/*z*): [M + Na]^+^ calcd. for C_40_H_46_O_2_Na, 581.3390; found, 581.3392.

### Synthesis of 7

Burgess reagent (180 mg, 0.754 mmol) was added to a solution of 6 (175 mg, 0.314 mmol) in THF (7.0 mL). After stirring for 1 h at 65 °C, the reaction was quenched by pouring water. The combined organic was extracted with EtOAc and washed with water. After drying over with MgSO_4_, the solvent was removed under reduced pressure and the residue was purified by column chromatography on silica gel (hexane: CHCl_3_ = 1: 1) to give 7 (110 mg, 67%) as a brown solid. ^1^H NMR (400 MHz, CDCl_3_, TMS): *δ* 0.90 (t, *J* = 6.9 Hz, 6 H), 1.28–1.42 (m, 12 H), 1.64–1.74 (m, 4 H), 2.70 (t, *J* = 7.3 Hz, 4 H), 3.76 (d, *J* = 7.3 Hz, 4 H), 5.95–6.04 (m, 2 H), 6.46–6.53 (m, 2 H), 7.14–7.19 (m, 4 H), 7.59 (d, *J* = 1.4 Hz, 2 H), 7.98 (d, *J* = 7.8 Hz, 2 H); ^13^C NMR (150 MHz, CDCl_3_): *δ* 14.1, 22.7, 29.0, 29.4, 31.6, 31.8, 36.1, 120.1, 122.3, 122.8, 122.9, 123.8, 127.4, 127.9, 133.3, 137.3, 140.9, 141.2, 141.3, 141.9; HRMS (*m*/*z*): [M + H]^+^ calcd. for C_40_H_43_, 523.3359; found, 523.3353.

### Synthesis of 2

To a solution of 7 (22 mg, 0.042 mmol) in CH_2_Cl_2_ (3.2 mL), a solution of DDQ (9.6 mg, 0.042 mmol) in CH_2_Cl_2_ (1.0 mL) was added slowly at −40 °C. After stirring for 20 min, the reaction was allowed to warm to ambient temperature. The solvent was removed under reduced pressure and the residue was washed with acetone to give 2 (11 mg, 50%) as a dark green solid. Recrystallization from hexane/CHCl_3_ gave a pure compound. mp: 233–235 °C; ^1^H NMR (400 MHz, CDCl_3_, TMS): *δ* 0.89 (t, *J* = 6.9 Hz, 6 H), 1.27–1.38 (m, 12 H), 1.64–1.72 (m, 4 H), 2.75 (t, *J* = 7.6 Hz, 4 H), 5.92–5.97 (m, 2 H), 6.46–6.51 (m, 2 H), 7.02 (d, *J* = 11.5 Hz, 2 H), 7.28–7.34 (m, 4 H), 7.66 (s, 2 H), 7.80 (d, *J* = 8.7 Hz, 2 H); ^13^C NMR (175 MHz, CDCl_3_): *δ* 14.2, 22.6, 29.1, 31.8, 31.9, 36.2, 119.5, 120.7, 123.6, 126.0, 128.8, 129.0, 133.0, 134.4, 134.6, 136.5, 138.0, 140.4, 149.1, 150.6; UV/vis (CH_2_Cl_2_): *λ*_max_ (*ε* [M^−1^ cm^−1^]) = 279 (55000), 304 (51000), 498 (36000), 692 (26000); HRMS (*m*/*z*): [M + H]^+^ calcd. for C_40_H_41_, 521.3203; found, 521.3196.

## Electronic supplementary material

supplementary information

## References

[1] Bendikov M, Wudl F, Perepichka DF (2004). Tetrathiafulvalenes, oligoacenenes, and their buckminsterfullerene derivatives: the brick and mortar of organic electronics. Chem. Rev..

[2] Anthony JE (2006). Functionalized acenes and heteroacenes for organic electronics. Chem. Rev..

[3] Anthony JE (2008). The larger acenes: versatile organic semiconductors. Angew. Chem. Int. Ed..

[4] Zade SS, Bendikov M (2010). Heptacene and beyond: the longest characterized acenes. Angew. Chem. Int. Ed..

[5] Li J, Zhang Q (2013). Mono- and oligocyclic aromatic ynes and diynes as building blocks to approach larger acenes, heteroacenes, and twistacenes. Synlett..

[6] Watanabe M, Chen K-Y, Chang YJ, Chow T (2013). Acenes generated from precursors and their semiconducting properties. Acc. Chem. Res..

[7] Ye Q, Chi C (2014). Recent highlights and perspectives on acene based molecules and materials. Chem. Mater..

[8] Randić M (2003). Aromaticity of polycyclic conjugated hydrocarbons. Chem. Rev..

[9] Kertesz M, Choi CH, Yang S (2005). Conjugated polymers and aromaticity. Chem. Rev..

[10] Hopf H (2013). Pentalenes−from highly reactive antiaromatics to substrates for material science. Angew. Chem. Int. Ed..

[11] Breslow R (2014). Novel aromatic and antiaromatic systems. Chem. Rec..

[12] Blood, C. T. & Linstead, R. P. Fused carbon rings. Part XXI. Dibenzopentalene. *J*. *Chem*. *Soc*. 2263–2268 (1952).

[13] Chuen CC, Fenton SW (1958). Dibenzopentalene. J. Org. Chem..

[14] Brown R, Eastwood F, Wong N (1993). The ethyne-ethylidene rearrangement: formation of indeno[2,1-*a*]indene and fluoranthene on flash vacuum pyrolysis of 1,4-diphenylbutadiyne. Tetrahedron Lett..

[15] Preda DV, Scott LT (2000). Phenyl migrations in dehydroaromatic compounds. A new mechanistic link between alternant and nonalternant hydrocarbons at high temperatures. Org. Lett..

[16] Kendall JK, Shechter H (2001). Intramolecular behaviors of anthryldicarbenic systems: dibenzo[*b*,*f*]pentalene and 1*h*,5*h*-dicyclobuta[*de*,*kl*]anthracene. J. Org. Chem..

[17] Babu G, Orita A, Otera J (2008). Facile carbolithiation of bent alkyne without catalyst. Tandem route to dibenzo[*b*,*f*]pentalenes from dibenzocyclooctadiyne. Chem. Lett..

[18] Kawase T (2009). An extremely simple dibenzopentalene synthesis from 2-bromo-1-ethynylbenzenes using nickel(0) complexes: construction of its derivatives with various functionalities. Chem. Eur. J..

[19] Levi ZU, Tilley TD (2009). Versatile synthesis of pentalene derivatives via the Pd-catalyzed homocoupling of haloenynes. J. Am. Chem. Soc..

[20] Kawase T (2010). Dinaphthopentalenes: pentalene derivatives for organic thin-film transistors. Angew. Chem. Int. Ed..

[21] Takahashi K, Ito S, Shintani R, Nozaki K (2017). Selective synthesis of unsymmetric dibenzo[*a*,*e*]pentalenes by a rhodium-catalysed stitching reaction. Chem. Sci..

[22] Willner I, Rabinovitz M (1978). 1,9-Dimethyldibenzo[*b*,*f*]pentalene dication and dianion. New 14π and 18π aromatic systems. J. Am. Chem. Soc..

[23] Willner I, Becker JY, Rabinovitz M (1979). Manifestation of dual aromaticity in doubly charged annelated pentalenes. J. Am. Chem. Soc..

[24] Saito M, Nakamura M, Tajima T, Yoshioka M (2007). Reduction of phenyl silyl acetylenes with lithium: unexpected formation of a dilithium dibenzopentalenide. Angew. Chem. Int. Ed..

[25] Saito M, Nakamura M, Tajima T (2008). New reactions of a dibenzo[*a*,*e*]pentalene. Chem. Eur. J..

[26] Kuwabara T, Ishimura K, Sasamori T, Tokitoh N, Saito M (2014). Facile synthesis of dibenzopentalene dianions and their application as new π-extended ligands. Chem. Eur. J..

[27] Oshima H, Fukazawa A, Yamaguchi S (2017). Facile synthesis of polycyclic pentalenes with enhanced hückel antiaromaticity. Angew. Chem. Int. Ed..

[28] Konishi A (2017). Synthesis and characterization of dibenzo[*a*,*f*]pentalene: harmonization of the antiaromatic and singlet biradical character. J. Am. Chem. Soc..

[29] Chase DT, Rose BD, McClintock SP, Zakharov LN, Haley MM (2011). Indeno[1,2-*b*]fluorenes: fully conjugated antiaromatic analogues of acenes. Angew. Chem. Int. Ed..

[30] Chase DT (2011). Electron-accepting 6,12-diethynylindeno[1,2-*b*]fluorenes: synthesis, crystal structures, and photophysical properties. Angew. Chem. Int. Ed..

[31] Chase DT (2012). 6,12-Diarylindeno[1,2-*b*]fluorenes: syntheses, photophysics, and ambipolar OFETs. J. Am. Chem. Soc..

[32] Young BS (2014). Synthesis and properties of fully-conjugated indacenedithiophenes. Chem. Sci..

[33] Frederickson CK, Zakharov LN, Haley MM (2016). Modulating paratropicity strength in diareno-fused antiaromatics. J. Am. Chem. Soc..

[34] Dressler JJ (2017). M. M. Synthesis of the unknown indeno[1,2-*a*]fluorene regioisomer: crystallographic characterization of its dianion. Angew. Chem. Int. Ed..

[35] Shimizu A, Tobe Y (2011). Indeno[2,1-*a*]fluorene: an air-stable ortho-quinodimethane derivative. Angew. Chem. Int. Ed..

[36] Shimizu A (2013). Indeno[2,1-*b*]fluorene: a 20-π-electron hydrocarbon with very low-energy light absorption. Angew. Chem. Int. Ed..

[37] Nishida J-I, Tsukaguchi S, Yamashita Y (2012). Synthesis, crystal structures, and properties of 6,12-diaryl-substituted indeno[1,2-. b]fluorenes. Chem. Eur. J..

[38] Tobe, Y. Non-alternant non-benzenoid aromatic compounds: past, present, and future. *Chem*. *Rec*. **15**, 86–96 (2015). 10.1002/tcr.201402077 25371318

[39] Frederickson CK, Rose BD, Haley MM (2017). Explorations of the indenofluorenes and expanded quinoidal analogues. Acc. Chem. Res..

[40] Oth JFM, Müllen K, Königshofen H, Wassen J, Vogel E (1974). The dianion of heptalene. Helv. Chim. Acta..

[41] Sugihara Y, Saito J, Murata I (1991). 2-Triphenylmethyldicyclohept[*cd*,*g*]indene: a novel *cata-peri* condensed nonalternant hydrocarbon. Angew. Chem. Int. Ed..

[42] Murata I (1993). Novel bonding structure of some nonalternant polycyclic systems. Pure Appl. Chem..

[43] Sugihara Y, Saito J, Murata I (1992). [14π] Electrocyclization followed by preferential [1,9]hydrogen migration in the thermolysis of 1-formyl-4-[(2,4,6-cycloheptatrienyl)(phenylsulfinyl)methyl]azulene. Bull. Chem. Soc. Jpn..

[44] Yang X, Liu D, Miao Q (2014). Heptagon-embedded pentacene: synthesis, structures, and thin-film transistors of dibenzo[*d*,*d’*]benzo[1,2-*a*:4,5-*a’*]dicycloheptenes. Angew. Chem. Int. Ed..

[45] Xu X (2015). Solution-processed ambipolar organic thin-film transistors by blending p- and n-type semiconductors: solid solution versus microphase separation. ACS Appl. Mater. Interfaces.

[46] Yang X (2016). Benzo[4,5]cyclohepta[1,2-*b*]fluorene: an isomeric motif for pentacene containing linearly fused five-, six- and seven-membered rings. Chem. Sci..

[47] Cheung KY, Xu X, Miao Q (2015). Aromatic saddles containing two heptagons. J. Am. Chem. Soc..

[48] Müller E, Sauerbier M, Heiß J (1966). Ein beitrag zum cyclobutadienproblem. Tetrahedaron Lett..

[49] Fischer H, Ege G (1967). Quantenchemische berechnungen als beitrag zur konstitutionsaufklarung kondensierter azulensysteme. Chem. Ber..

[50] Müller E (1970). Zur struktur des verdens. Liebigs Ann. Chem..

[51] Scholl M, Ding S, Lee CW, Grubbs RH (1999). Synthesis and activity of a new generation of ruthenium-based olefin metathesis catalysts coordinated with 1,3-dimesityl-4,5-dihydroimidazol-2-ylidene ligands. Org. Lett..

[52] Burgess E, Penton HR, Taylor EA (1973). Thermal reactions of alkyl *N*-carbomethoxysulfamate esters. J. Org. Chem..

[53] Ito S, Minami T, Nakano M (2012). Diradical character based design for singlet fission of condensed-ring systems with 4*n*π electrons. J. Phys. Chem. C.

[54] Bard, A. J. & Faulkner, L. R. *Electrochemical methods-fundamentals and applications*. Wiley, New York (1984).

[55] Pommerehne J (1995). Efficient two layer LEDs on a polymer blend basis. Adv. Mater..

[56] Cardona CM, Li W, Kaifer AE, Stockdale D, Bazan GC (2011). Electrochemical considerations for determining absolute frontier orbital energy levels of conjugated polymers for solar cell applications. Adv. Mater..

[57] Kolc J, Downing JW, Manzara AP, Michl J (1976). π, π-Biradicaloid hydrocarbons. The pleiadene family. II. A doubly exited state of pleiadene. J. Am. Chem. Soc..

[58] Di Motta S, Negri F, Fazzi D, Castiglioni C, Canesi EV (2010). Biradicaloid and polyenic character of quinoidal oligothiophenes revealed by the presence of a low-lying double-exciton state. J. Phys. Chem. Lett..

[59] Kruszewski J, Krygowski TM (1972). Definition of aromaticity basing on the harmonic oscillator model. Tetrahedron Lett..

[60] Poater J, Duran M, Solà M, Bernard Silvi B (2014). Theoretical Evaluation of Electron Delocalization in Aromatic Molecules by Means of Atoms in Molecules (AIM) and Electron Localization Function (ELF) Topological Approaches. Chem. Rev..

[61] Gershoni-Poranne R, Stanger A (2014). The NICS-*XY*-scan: identification of local and global ring currents in multi-ring systems. Chem. Eur. J..

[62] Herges R, Geuenich D (2001). Delocalization of Electrons in Molecules. J. Phys. Chem. A.

