# Supplementary material for: Antiaromatic character of cycloheptatriene-bis-annelated indenofluorene framework mainly originated from heptafulvene segment
Source: Sci Rep. 2018 Dec 5;8:17663. doi: 10.1038/s41598-018-35839-w (PMC6281641; doi:10.1038/s41598-018-35839-w)
Supplement: Supplementary file 1 — supplementary information [file 41598_2018_35839_MOESM1_ESM.docx]

**Electronic Supplementary Information**

**Antiaromatic character of cycloheptatriene-bis-annelated indenofluorene framework mainly originated from heptafulvene segment**

Keitaro Yamamoto,^a^ Yutaka Ie, ^a,b,^* Norimitsu Tohnai,^c^ Fumitoshi Kakiuchi,^b,d^ and Yoshio Aso ^a,^*

*^a^ The Institute of Scientific and Industrial Research (ISIR), Osaka University, 8-1 Mihogaoka, Ibaraki, Osaka 567-0047, Japan*

^b^ Japan Science and Technology (JST) Agency, ACT-C, 4-1-8 Honcho, Kawaguchi, Saitama 332-0012, Japan.

*^c^ Department of Materials and Life Science, Graduate School of Engineering, Osaka University, 2-1 Yamadaoka, Suita, Osaka 565-0871, Japan*

*^d^ Department of Chemistry, Faculty of Science and Technology, Keio University, 3-14-1 Hiyoshi, Kohoku-ku, Yokohama, Kanagawa 223-8522, Japan*

**Table of Contents**

**Supplementary figures S2-S4**

**Synthesis S5-S6**

**X-ray information S7**

**OFET device fabrication S7**

**Computational details S8-S11**

**References S11**

**Supplementary figures**


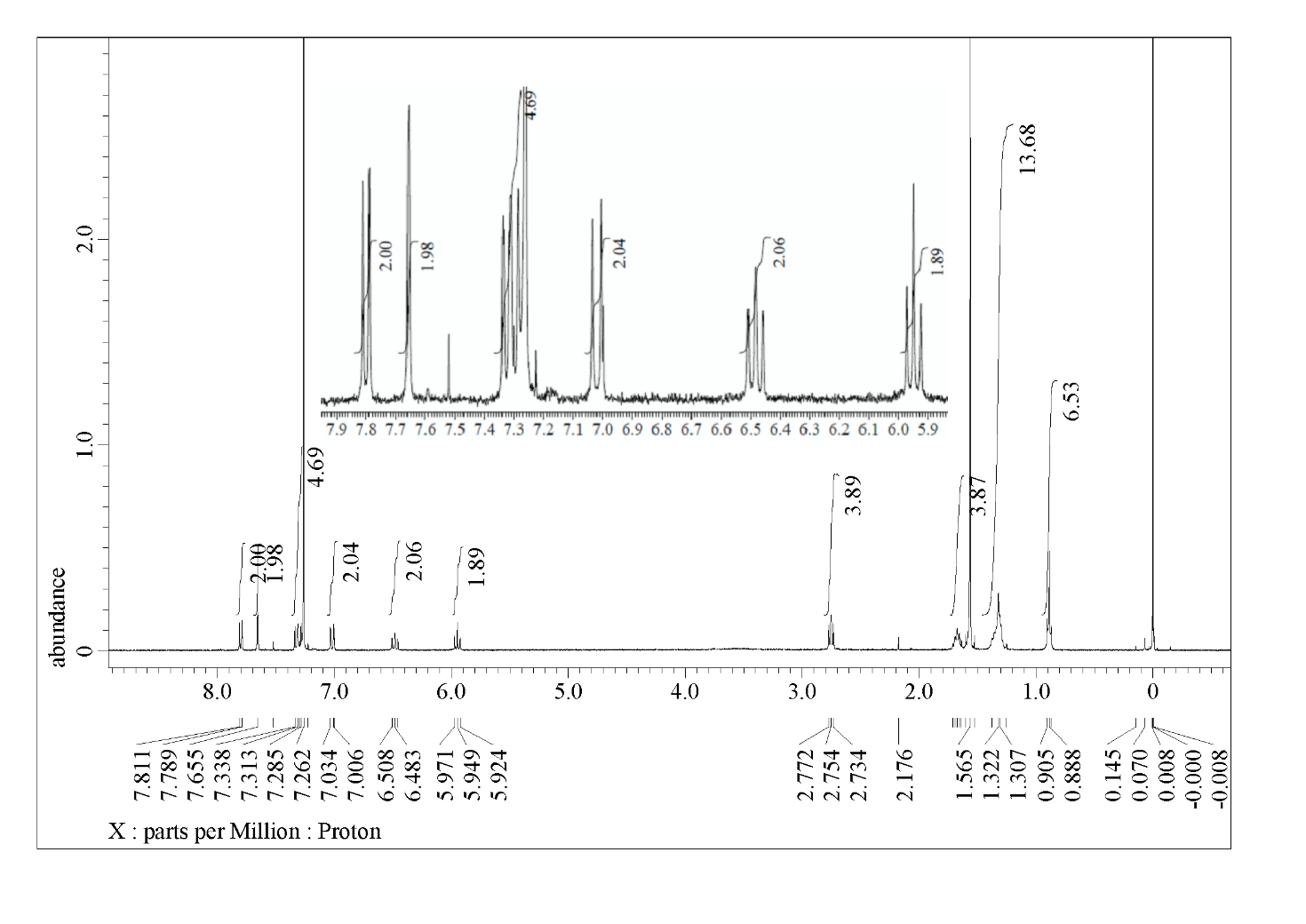


**
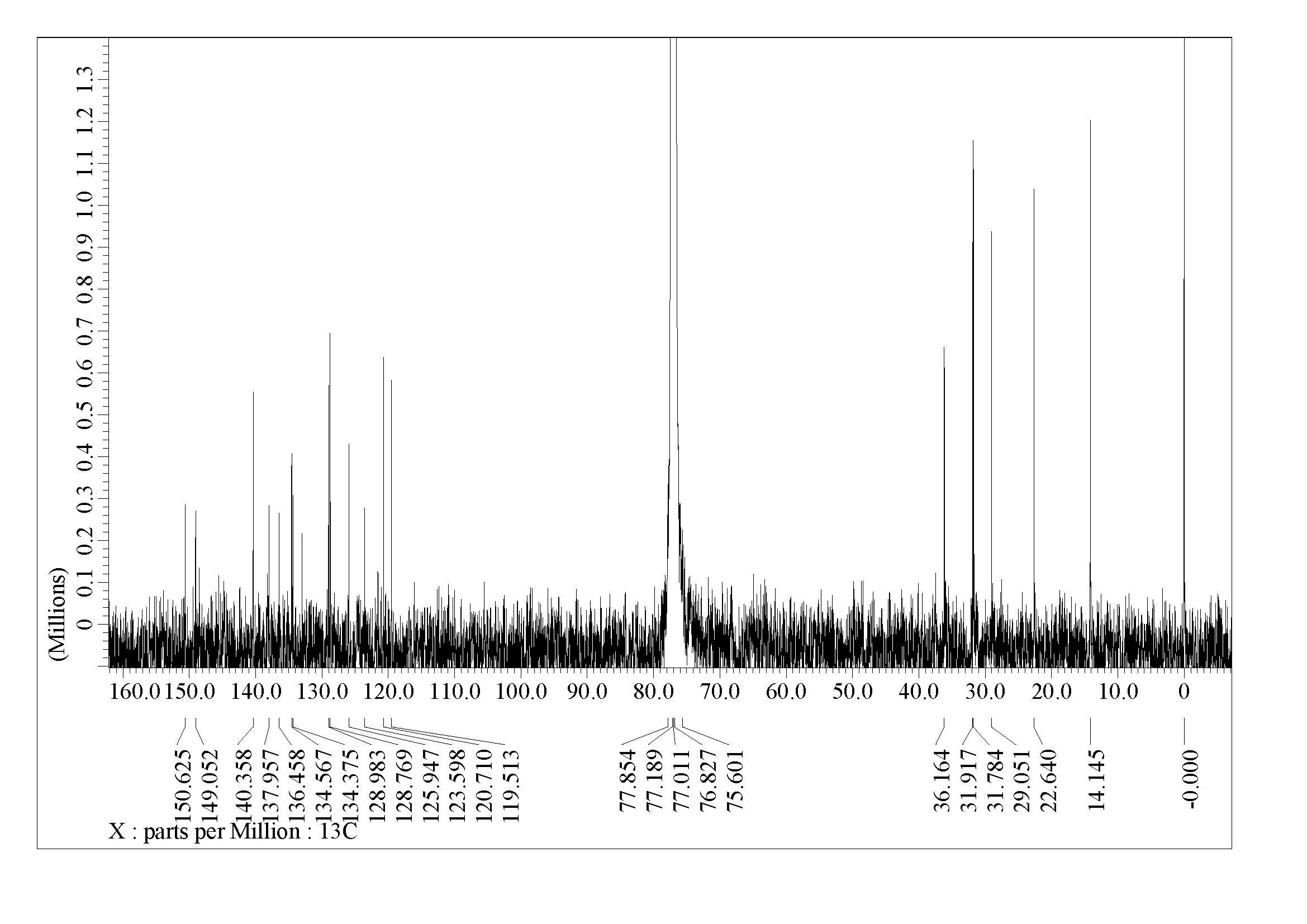
**

**Supplementary Fig. 1** ^1^H NMR (400 MHz) and ^13^C NMR (150 MHz) spectra of **2** in CDCl3.


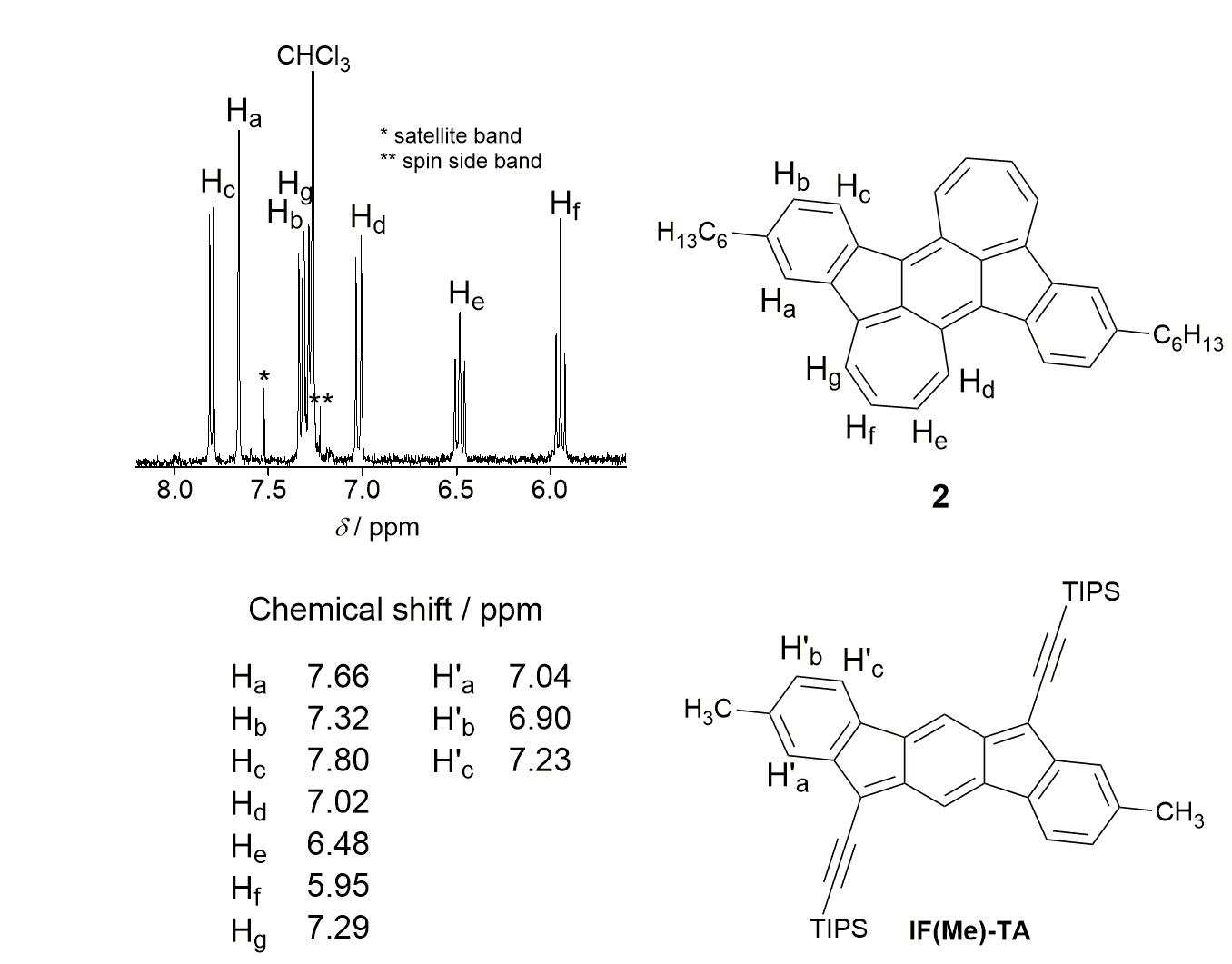


**Supplementary Fig. 2** ^1^H NMR spectrum of **2** in aromatic regions in CDCl_3_. Chemical shifts of **IF(Me)-TA** were extracted from reference 1 in the ESI.


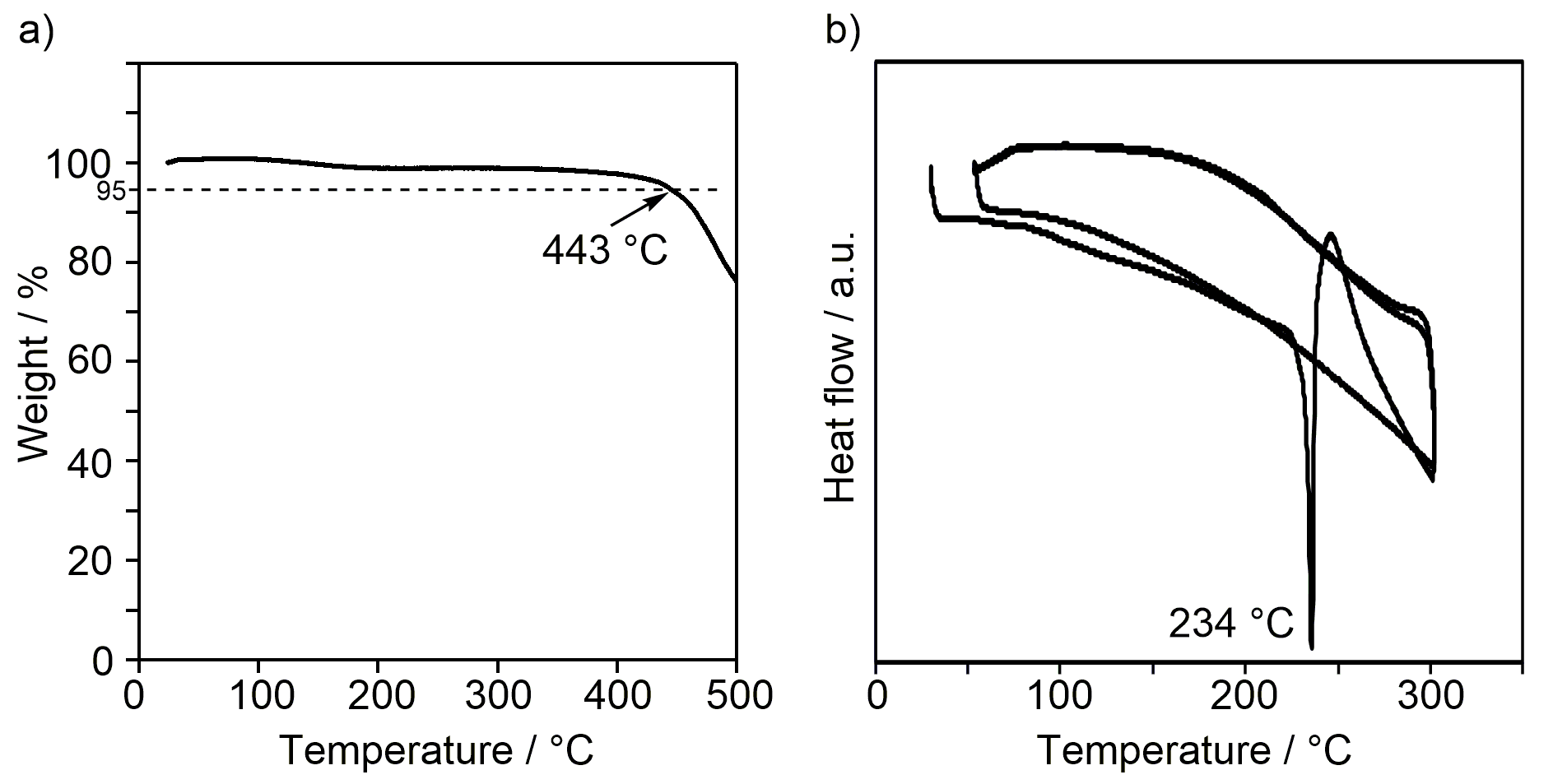


**Supplementary Fig. 3** (a) TGA and (b) DSC curves of **2** with a heating rate of 10 °C min^–1^ in N_2_.


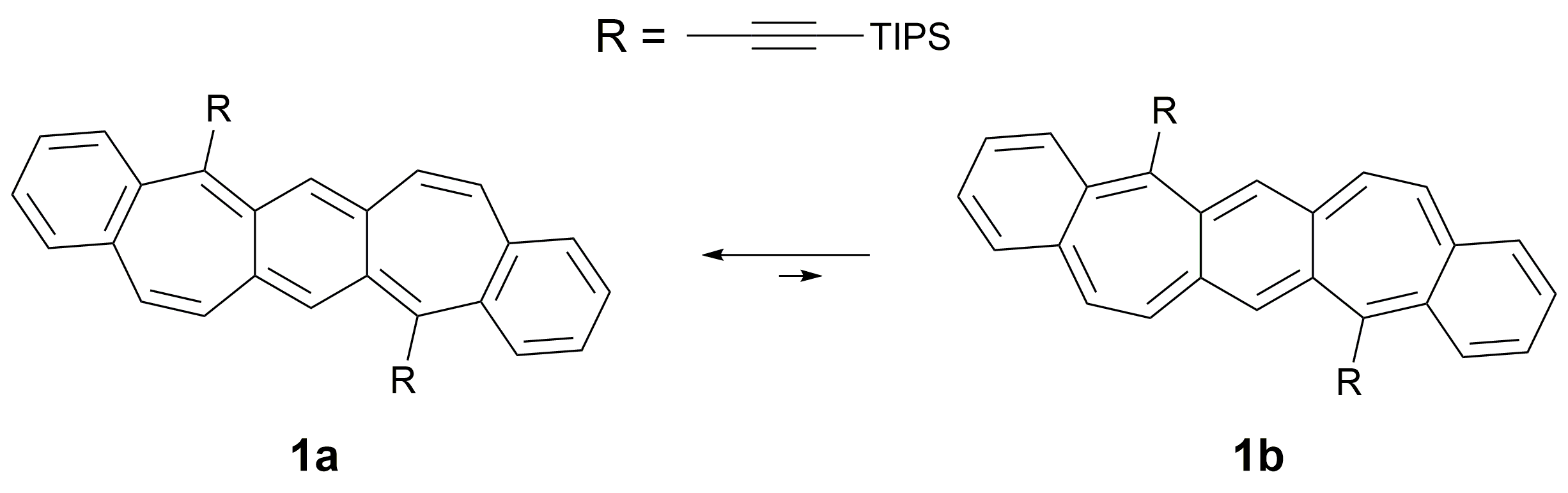


**Supplementary Fig. 4** Resonance chemical structures of **1**.


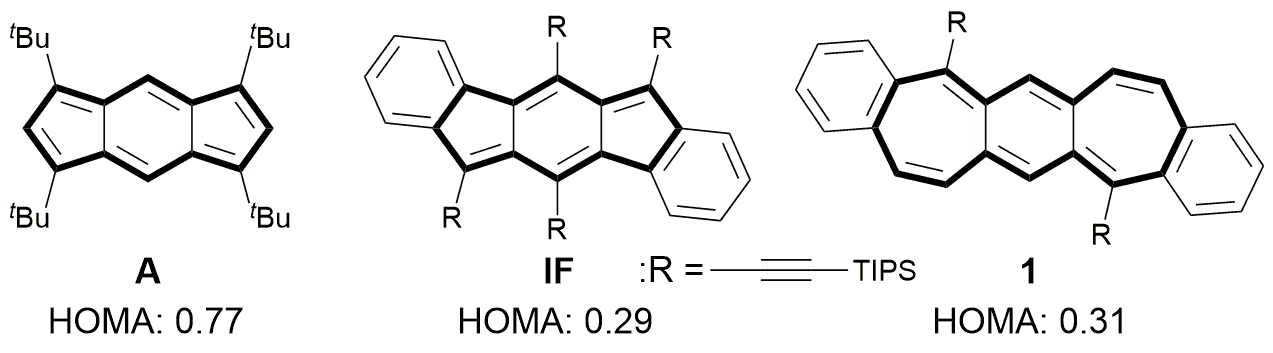


**Supplementary Fig. 5** HOMA values for **A**, **IF**, and **1**. X-ray crystallographic data for **A**, **IF**, and **1** are obtained from references 2, 3, and 4, respectively**.**


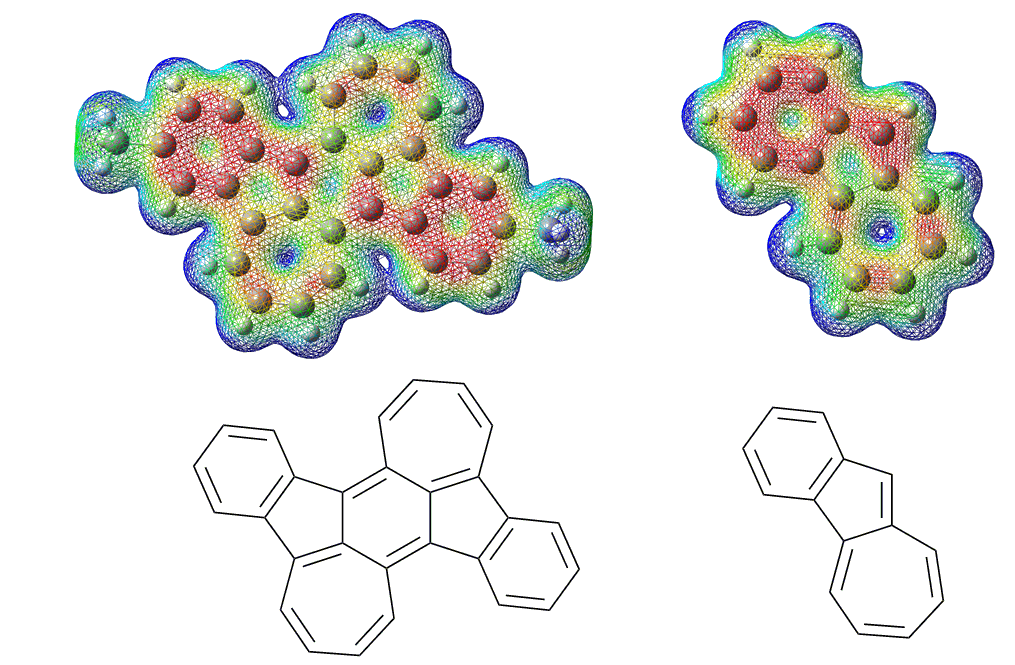


**Supplementary Fig. 6** Electrostatic potentials of **2(H)** (left) and benz[*a*]azulene (right).


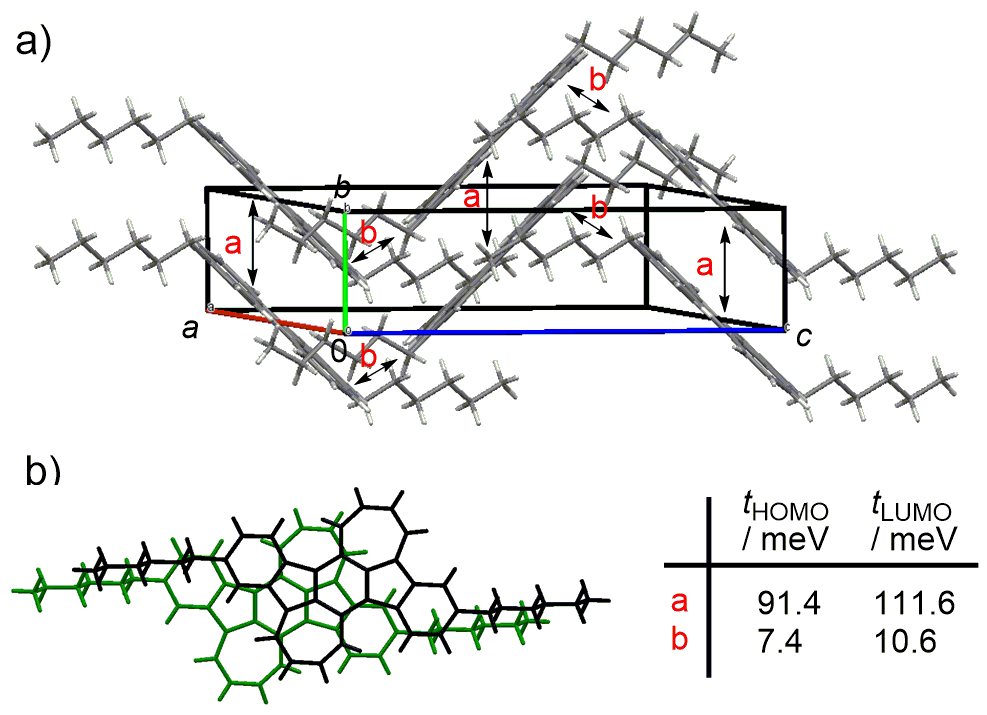


**Supplementary** **Fig. 7** (a) Packing diagram and (b) estimated transfer integrals of HOMOs and LUMOs for **2**.

_
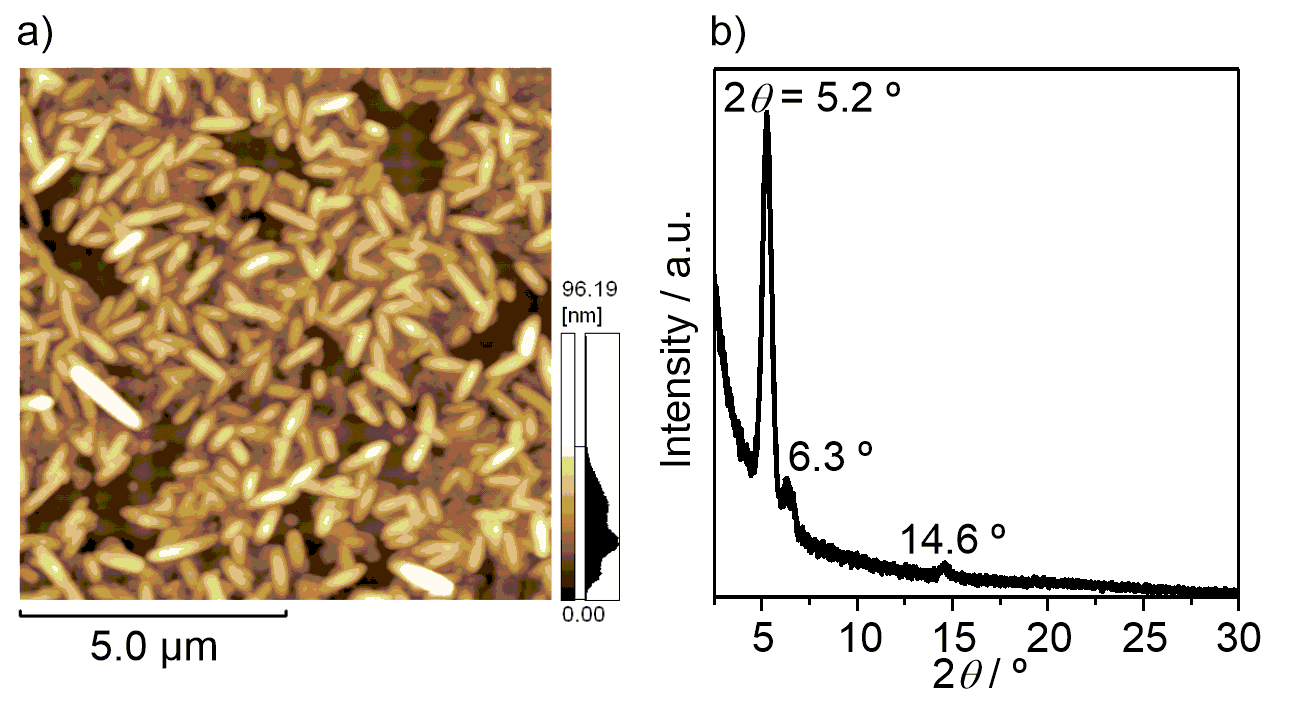
_

**Supplementary Fig. 8** a) AFM height image and b) XRD data of **2**.

**Synthesis**

Unless stated otherwise, all reagents were purchased from commercial sources and used without purification. Compound **s-1** was prepared by the reported procedure.^5^


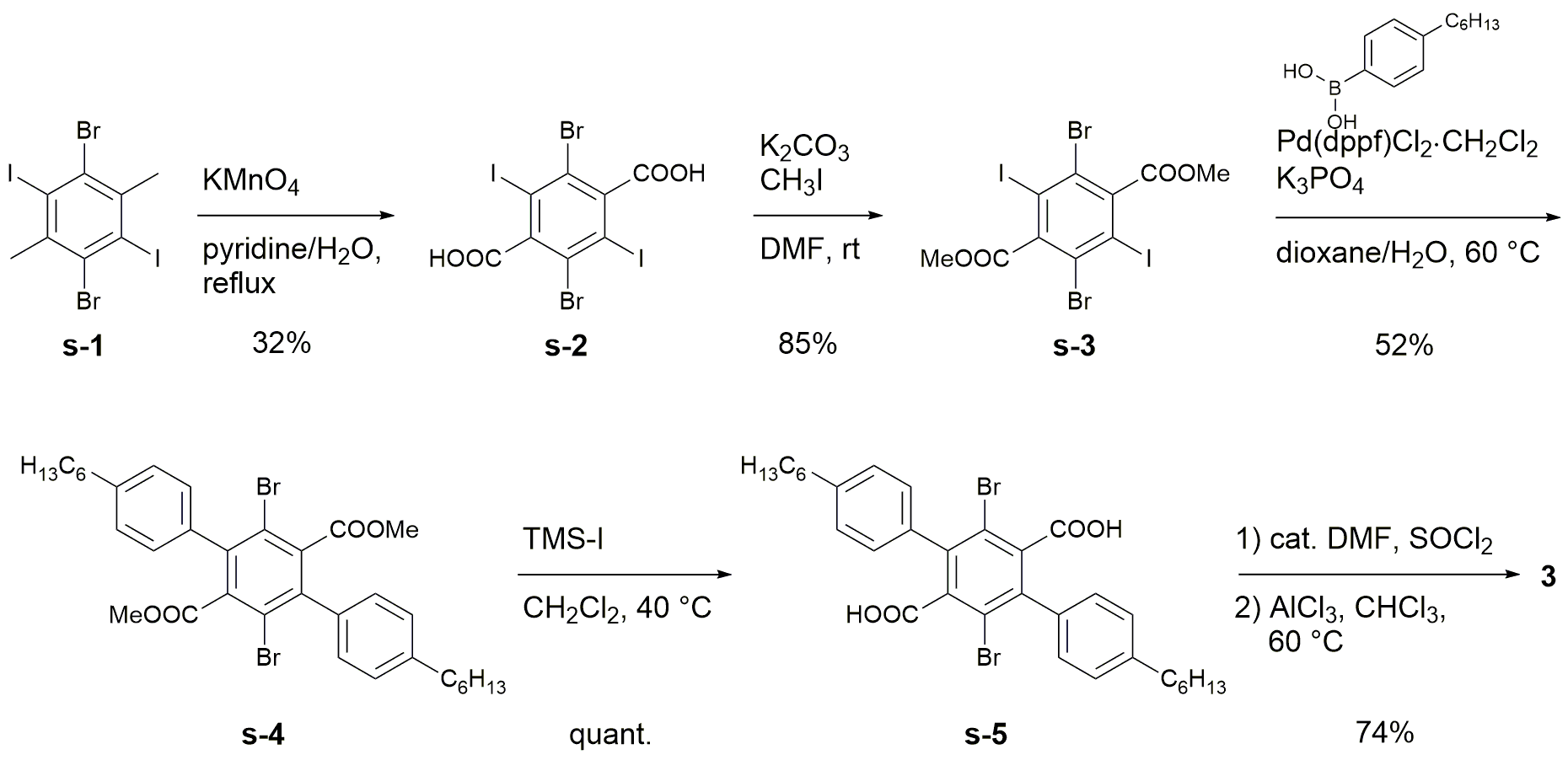


**Supplementary Fig. 9** Synthetic route of **3**.

*Synthesis of* ***s-2***: Potassium permanganate (42.6 g, 270 mmol) was added in six portions to a solution of compound **s-1** (6.96 g, 13.5 mmol) in pyridine (242 mL) and water (71 mL), and the mixture was stirred for overnight at reflux condition. The reaction mixture was passed through pad of celite under hot condition to remove residue of potassium permanganate. The filtrate was washed with ethyl acetate (EtOAc) for two times, and the aqueous layer was acidified by 10% hydrochloric acid. The precipitate in the aqueous layer was extracted with EtOAc. After drying with MgSO_4_, the solvent was removed under reduced pressure. Then potassium hydroxide (4.04 g, 72.0 mmol) was added to the suspension of obtained solid in water (216 mL), and the mixture was allowed to warm to 90 °C. To this solution, potassium permanganate (13.0 g, 82.3 mmol) was added, and this reaction mixture was stirred for 6 h. After completion of the reaction, methanol was added to the reaction mixture at room temperature in order to quench the unreacted potassium permanganate. Then, this mixture was passed through pad of celite under hot condition. The filtrate was acidified by 2N hydrochloric acid, and the precipitate was extracted with EtOAc. After drying with MgSO_4_, the solvent was removed under reduced pressure to give **s-2** (2.51 g, 32%) as a white solid. ^1^H NMR (400 MHz, DMSO-*d*_6_): *δ* 14.30 (br, 2H); ^13^C NMR (100 MHz, DMSO-*d*_6_): *δ* 103.5, 126.2, 146.5, 168.6. This compound was used for next step without further purification.

*Synthesis of* ***s-3***: Compound **s-2** (2.49 g, 4.33 mmol), potassium carbonate (2.39 g, 17.3 mmol) in DMF (10.4 mL) was stirred for 10 min at room temperature. Then, iodomethane (1.08 mL, 17.3 mmol) was added to this mixture. After stirring for 1 h at 35 °C, water was poured into the reaction mixture. The resulting mixture was extracted with EtOAc. The organic layer was washed with water and dried over MgSO_4_. The solvent was removed under vacuum condition to give **s-3** (2.21 g, 85%) as a white solid. ^1^H NMR (400 MHz, CDCl_3_, TMS): *δ* 3.99 (s, 6H); ^13^C NMR (150 MHz, CDCl_3_): *δ* 54.0, 101.4, 126.7, 145.1, 166.9; HRMS (m/z): [M+H]^+^ calcd. for C_10_H_7_Br_2_I_2_O_4_, 604.6775; found, 604.6760.

*Synthesis of* ***s-4***: Compound **s-3** (2.21 g, 3.66 mmol), 4-hexylphenylboronic acid (2.26 g, 11.0 mmol), Pd(dppf)Cl_2_∙CH_2_Cl_2_ (59.8 mg, 0.0732 mmol), and K_3_PO_4_ (3.11 g, 14.6 mmol), 1,4-dioxance (51 mL), and water (14 mL) were added to a round-bottom flask. This flask was purged with N_2_. After stirring for 16 h at 60 °C, the mixture was extracted with EtOAc, and the organic layer was washed with water. After drying with MgSO_4_, the solvent was removed under reduced pressure. The residue was purified by column chromatography on silica gel (CHCl_3_), followed by purification with preparative GPC (CHCl_3_) to give **s-4** (1.28 g, 52%) as a white solid. ^1^H NMR (400 MHz, CDCl_3_, TMS): *δ* 0.89 (t, *J* = 7.0 Hz, 6H), 1.27-1.38 (m, 12H), 1.60-1.70 (m, 4H), 2.65 (t, *J* = 7.8 Hz, 4H), 3.53 (s, 6H), 7.17-7.25 (m, 8H); ^13^C NMR (150 MHz, CDCl_3_): *δ* 14.4, 23.0, 29.3, 31.5, 32.0, 36.1, 52.8, 120.2, 128.5, 129.5, 135.0, 139.2, 141.5, 143.8, 167.0; HRMS (m/z): [M+Na]^+^ calcd. for C_34_H_40_Br_2_O_4_Na, 695.1165; found, 695.1168.

*Synthesis of* ***s-5***: To a solution of **s-4** (1.28 g, 1.90 mmol) in CH_2_Cl_2_ (14.4 mL), trimethylsilyl iodide (1.55 mL, 11.4 mmol) was added. After stirring for overnight at 40 °C, water was poured into the mixture at room temperature. The resultant mixture was extracted with EtOAc, and the organic layer was washed with water. After drying with MgSO_4_, the solvent was removed under reduced pressure to give **s-5** (1.22 g, quant.) as a white solid. ^1^H NMR (400 MHz, DMSO-*d*_6_, TMS): *δ* 0.89 (t, *J* = 6.8 Hz, 6H), 1.26-1.38 (m, 12H), 1.56-1.66 (m, 4H), 2.62 (t, *J* = 7.6 Hz, 4H), 7.19 (d, *J* = 8.4 Hz, 4H), 7.29 (d, *J* = 8.4 Hz, 4H), 13.49 (br, 2H). This compound was used for next step without further purification.

*Synthesis of* ***3***: Compound **s-5** (1.22 g, 1.89 mmol) was placed in a round-bottom flask. Thionyl chloride (23 mL) and DMF as a catalyst was added to the flask, and the reaction mixture was refluxed for 2 h. Then, thionyl chloride and DMF were removed under reduced pressure. The residue was resolved in CHCl_3_ (68 mL), and aluminum chloride (2.52 g, 18.9 mmol) was added to the solution. After stirring for 5 h at 60 °C, 10% hydrochloric acid was slowly poured into the reaction mixture at 0 °C. The resultant mixture was extracted with CHCl_3_, and the organic layer was washed with water and NaHCO_3_. After drying with MgSO_4_, the solvent was removed under reduced pressure, and the residue was washed with acetone to give **3** (850 mg, 74%) as a magenta solid; ^1^H NMR (600 MHz, CDCl_3_, TMS): *δ* 0.89 (t, *J* = 6.3 Hz, 6H), 1.27-1.38 (m, 12H), 1.60-1.67 (m, 4H), 2.66 (t, *J* = 8.1 Hz, 4H), 7.38 (d, *J* = 7.2 Hz, 2H), 7.54 (s, 2H), 8.49 (d, *J* = 7.2 Hz, 2H); ^13^C NMR (150 MHz, CDCl_3_): *δ* 14.1, 22.6, 28.8, 31.0, 31.6, 35.7, 114.4, 124.3, 124.7, 134.3, 135.1, 137.1, 138.8, 145.8, 146.7, 190.0; HRMS (m/z): [M+H]^+^ calcd. for C_32_H_33_Br_2_O_2_, 609.0822; found, 609.0818; analysis (calcd., found for C_32_H_32_Br_2_O_2_): C (63.17, 63.06), H (5.30, 5.43).

**X-ray information**

The diffraction data were collected on a Rigaku XtaLAB P200 diffractometer with monochromated CuKα (λ = 1.54187 Å) radiation. The structures were determined by a direct method (SHELXT Version). The non-hydrogen atoms were anisotropically refined. CCDC-1816207 contains the supplementary crystallographic data for **2**. These data can be obtained free of charge from The Cambridge Crystallographic Data Centre via www.ccdc.cam.ac.uk/data_request/cif/.

Crystal Data for **2**: C_40_H_40_, *M* = 520.76, monoclinic, space group *P*2_1_*/c* (#14), *a* = 18.0409(11), *b* = 4.7749(3), *c* = 17.2744(10) Å, *β* = 104.994(6)°, *V* = 1437.41(16) Å^–3^, *D*_calc_ = 1.203 g cm^–3^*, F*_(000)_ *=* 560.00*,* μ = 5.042 cm ^–1^ (CuKα), *Z* = 2, *R* = 0.063, *R_w_* = 0.1903 for all data.

**OFET device fabrication**

The field-effect electron mobility was measured using bottom-gate bottom-contact OFET devices. The p-doped silicon substrate functions as the gate electrode. A thermally grown silicon oxide (SiO2) dielectric layer on the gate substrate has 300 nm thick and a capacitance (*C*_i_) of 10.0 nF cm^–2^. Interdigital source and drain electrodes were constructed with gold (30 nm) that were formed on the SiO2 layer. The channel width (*W*) and channel length (*L*) are 38 mm and 5 μm, respectively. The silicon oxide surface was first washed with toluene, acetone, purified water and 2-propanol. It was then activated by ozone treatment and pretreated with HMDS. The semiconductor layer was vacuum-deposited on the HMDS-modified Si/SiO_2_ substrate at a rate of 0.1 Å s^–1^ under a pressure of 10^–6^ Pa to a thickness of 25 nm determined by a quartz crystal monitor. The characteristics of the OFETs were measured at room temperature under a pressure of 10^–3^ Pa by using a KEITHLEY 4200 semiconductor parameter analyzer. The hole mobility (*μ*_h_) was calculated in the saturated region at the *V*_DS_ of –80 V by the following equation.

Current on/off ratio was determined from the *I*DS at *V*GS= 0 V (*I*off) and *V*GS= –80 V (*I*on)

**Computational details**

All calculations were conducted using Gaussian 09 program. The geometry was optimized with the restricted Becke Hybrid (B3LYP) at 6-311+G(d,p) level. The time-dependent density functional theory (TD-DFT) calculations were conducted at the CAM-B3LYP/6-31G(d,p) level of theory. As shown below, optimized structure of **2** reproduces X-ray crystal structure. However, to directly correlate with HOMA values, nucleus-independent chemical shift (NICS) calculations and biradical character calculations were conducted by the geometry obtained by X-ray crystal structure analysis.


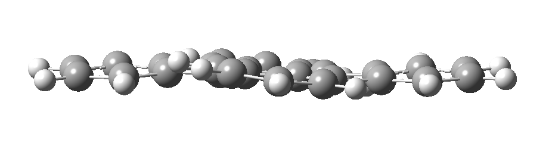

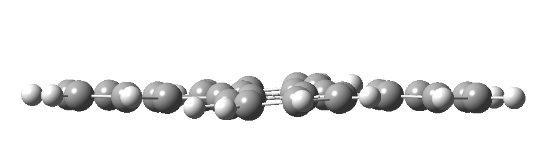


Side views of **2** for optimized chemical structure at B3LYP 6-311+G(d,p) level (left) and X-ray structure (right). For X-ray structure, alkyl groups were omitted for clarity.

The ELF was calculated with the DGrid 5.0 program,^6^ and the isosurface was visualized using the VESTA program.

TD-DFT calculation

**2(Me)**

Excited State 1: Singlet-A 2.03 eV 612 nm *f* = 0.29 <S**2>=0.000

HOMO–1 > LUMO+1 0.23678

HOMO > LUMO 0.65657

Excited State 2: Singlet-A 2.98 eV 416 nm *f* = 0.14 <S**2>=0.000

HOMO–3 > LUMO+1 0.10330

HOMO –2> LUMO 0.27783

HOMO–1> LUMO+1 0.38943

HOMO > LUMO 0.19034

HOMO > LUMO+2 0.45979

Excited State 3: Singlet-A 3.26 eV 381 nm *f* = 0.34 <S**2>=0.000

HOMO–1 > LUMO+1 0.51868

HOMO > LUMO 0.14208

HOMO> LUMO+2 0.43182

Optimized structure of **2(H)** at B3LYP/6-311+G(d,p).

--------------------------------------------------------------------------------------------------------

Center Atomic Atomic Coordinates (Angstroms)

Number Number Type X Y Z

--------------------------------------------------------------------------------------------------------

1 6 0 2.515055 1.296930 -0.150975

2 6 0 -2.518060 -1.288403 0.173742

3 6 0 5.481127 -1.032009 0.296162

4 6 0 4.819894 0.173813 0.122609

5 6 0 3.420271 0.191765 0.087251

6 6 0 3.356628 -2.217534 0.383177

7 6 0 4.746218 -2.221391 0.409985

8 6 0 2.660838 -1.002550 0.249299

9 6 0 1.246930 -0.638892 0.172902

10 6 0 1.176241 0.753164 -0.109636

11 6 0 -0.067013 1.427527 -0.326275

12 6 0 -1.249202 0.646704 -0.149830

13 6 0 -1.178779 -0.745571 0.131176

14 6 0 0.064561 -1.420474 0.346208

15 6 0 -2.662754 1.011746 -0.222510

16 6 0 -3.357139 2.228013 -0.351468

17 6 0 -4.746782 2.233591 -0.374603

18 6 0 -5.482742 1.044712 -0.261810

19 6 0 -4.822605 -0.162344 -0.092499

20 6 0 -3.422911 -0.182057 -0.060701

21 6 0 -0.155614 2.777949 -0.765086

22 6 0 2.944142 2.605027 -0.366952

23 6 0 2.190921 3.720522 -0.697971

24 6 0 0.806754 3.761139 -0.910581

25 6 0 -2.947863 -2.596531 0.387831

26 6 0 0.152705 -2.772667 0.779502

27 6 0 -0.810138 -3.755697 0.923322

28 6 0 -2.194754 -3.713466 0.714213

29 1 0 6.564222 -1.060373 0.324854

30 1 0 5.389863 1.088860 0.005234

31 1 0 2.847997 -3.168777 0.438106

32 1 0 5.270114 -3.165342 0.512134

33 1 0 -2.846908 3.178543 -0.404802

34 1 0 -5.269915 3.178378 -0.472849

35 1 0 -6.565874 1.074430 -0.287670

36 1 0 -5.393464 -1.076881 0.024549

37 1 0 -1.147337 3.097065 -1.042434

38 1 0 4.015476 2.766275 -0.289847

39 1 0 2.730472 4.649968 -0.845358

40 1 0 0.421740 4.720703 -1.246105

41 1 0 -4.019502 -2.756678 0.312670

42 1 0 1.144583 -3.094136 1.053118

43 1 0 -0.424977 -4.716790 1.254272

44 1 0 -2.734615 -4.642960 0.860148

--------------------------------------------------------------------------------------------------------

Optimized structure of **BDA** at B3LYP/6-311+G(d,p).

--------------------------------------------------------------------------------------------------------

Center Atomic Atomic Coordinates (Angstroms)

Number Number Type X Y Z

--------------------------------------------------------------------------------------------------------

1 6 0 2.562748 1.281506 -0.119228

2 6 0 -2.562805 -1.282257 0.116441

3 1 0 2.092745 -1.170928 0.496195

4 6 0 1.165616 -0.652328 0.275853

5 6 0 1.260184 0.702310 -0.078507

6 6 0 0.020443 1.395814 -0.373067

7 6 0 -1.165710 0.651686 -0.278234

8 6 0 -1.260273 -0.702971 0.076055

9 6 0 -0.020546 -1.396425 0.370803

10 1 0 -2.092837 1.170280 -0.498596

11 1 0 3.356757 0.585757 0.136563

12 1 0 -3.356782 -0.586612 -0.139729

13 6 0 -0.092150 2.766612 -0.749746

14 6 0 2.990585 2.563000 -0.424494

15 6 0 2.252451 3.691047 -0.791011

16 6 0 0.863734 3.752789 -0.929534

17 6 0 -2.990653 -2.563720 0.421829

18 6 0 0.092017 -2.767131 0.747833

19 6 0 -0.863874 -3.753284 0.927705

20 6 0 -2.252562 -3.691635 0.788837

21 1 0 -1.115027 3.087240 -0.925431

22 1 0 4.066770 2.706545 -0.368458

23 1 0 2.808068 4.601600 -0.985752

24 1 0 0.465891 4.720905 -1.223455

25 1 0 -4.066810 -2.707349 0.365480

26 1 0 1.114871 -3.087682 0.923789

27 1 0 -0.466064 -4.721305 1.221988

28 1 0 -2.808187 -4.602163 0.983671

--------------------------------------------------------------------------------------------------------

Optimized structure of **IF** at B3LYP/6-311+G(d,p).

--------------------------------------------------------------------------------------------------------

Center Atomic Atomic Coordinates (Angstroms)

Number Number Type X Y Z

--------------------------------------------------------------------------------------------------------

1 6 0 -1.164066 0.802160 0.006003

2 6 0 -1.233781 -0.664546 -0.002098

3 6 0 -0.031659 -1.448947 -0.006820

4 6 0 1.164066 -0.802166 -0.003621

5 6 0 1.233781 0.664539 0.004476

6 6 0 0.031659 1.448941 0.009203

7 6 0 2.549436 -1.286062 -0.006743

8 6 0 3.394171 -0.139142 -0.000664

9 6 0 2.554273 1.046257 0.006170

10 6 0 -2.549436 1.286056 0.009117

11 6 0 -3.394171 0.139136 0.003029

12 6 0 -2.554273 -1.046263 -0.003800

13 6 0 3.099101 -2.559992 -0.013980

14 6 0 4.492829 -2.696213 -0.015187

15 6 0 5.322744 -1.572124 -0.009229

16 6 0 4.780670 -0.284603 -0.001920

17 6 0 -3.099101 2.559986 0.016347

18 6 0 -4.492829 2.696207 0.017536

19 6 0 -5.322744 1.572118 0.011569

20 6 0 -4.780670 0.284597 0.004269

21 1 0 -0.096237 -2.532688 -0.012797

22 1 0 0.096237 2.532681 0.015181

23 1 0 2.920637 2.065141 0.011701

24 1 0 -2.920637 -2.065148 -0.009327

25 1 0 2.467802 -3.442345 -0.018665

26 1 0 4.933664 -3.686739 -0.020820

27 1 0 6.399141 -1.702191 -0.010299

28 1 0 5.429830 0.584607 0.002685

29 1 0 -2.467803 3.442339 0.021041

30 1 0 -4.933664 3.686733 0.023161

31 1 0 -6.399141 1.702185 0.012626

32 1 0 -5.429830 -0.584613 -0.000344

--------------------------------------------------------------------------------------------------------

**References**

(1) Chase, D. T., Fix, A. G., Rose, B. D., Weber, C. D., Nobusue, S., Stockwell, C. E., Zakharov, L. N., Lonergan, M. C. & Haley, M. M. Electron-accepting 6,12-diethynylindeno[1,2-*b*]fluorenes: synthesis, crystal structures, and photophysical properties. *Angew. Chem. Int. Ed.* **50**, 11103−11106 (2011).

(2) Dunitz, J. D., Krüger, C., Irngartinger, H., Maverick, E. F., Wang, Y. & Nixdorf, M. Equilibrium Structure, Stabilized Transition State, or Disorder in the Crystal? Studies of the Antiaromatic Systems Tetra-tert-butyl-s-indacene and Tetra-tert-butylcyclobutadiene by Low-Temperature Crystal Structure Analysis. *Angew. Chem. Int. Ed.* **27**, 387−389 (1988).

(3) Chase, D. T., Rose, B. D., McClintock, S. P., Zakharov, L. N. & Haley, M. M. Indeno[1,2-*b*]fluorenes: fully conjugated antiaromatic analogues of acenes. *Angew. Chem. Int. Ed.* **50**, 1127−1130 (2011).

(4) Yang, X., Liu, D. & Miao, Q. Heptagon-embedded pentacene: synthesis, structures, and thin-film transistors of dibenzo[*d*,*d*']benzo[1,2-*a*:4,5-*a*']dicycloheptenes. *Angew. Chem. Int. Ed.* **53**, 6786−6790 (2014).

(5) Rose, B. D., Santa Maria, P. J., Fix, A. G., Vonnegut, C. L., Zakharov, L. N., Parkin, S. R. & Haley, M. M. Scalable synthesis of 5,11-diethynylated indeno[1,2-*b*]fluorene-6,12-diones and exploration of their solid state packing. *Beilstein J. Org. Chem*. **10**, 2122−2130 (2014).

(6) Kohout, M. DGrid, version 5.0; Max Planck Institute for Chemical Physics of Solids: Dresden, Germany, 2017.
